# Global, Regional, and National Epidemiology of Vision Impairment due to Diabetic Retinopathy Among Working‐Age Population, 1990–2021

**DOI:** 10.1111/1753-0407.70121

**Published:** 2025-07-14

**Authors:** Yang Meng, Yuan Liu, Runping Duan, Baoyi Liu, Zhuangling Lin, Yuan Ma, Lan Jiang, Zijian Qin, Tao Li

**Affiliations:** ^1^ State Key Laboratory of Ophthalmology Zhongshan Ophthalmic Center, Sun Yat‐sen University, Guangdong Provincial Key Laboratory of Ophthalmology and Visual Science Guangzhou China; ^2^ Guangdong Provincial Clinical Research Center for Ocular Diseases Guangzhou China

**Keywords:** diabetic retinopathy, GBD, prediction, vision impairment, working‐age population

## Abstract

**Background:**

To evaluate the global, regional, and national trends of vision impairment associated with diabetic retinopathy (DR) in the working‐age population (20–65 years) from 1990 to 2021.

**Methods:**

This was a population‐based analysis using data from the Global Burden of Disease Study 2021. Vision impairment was defined as low vision (Snellen visual acuity of < 6/18 to ≥ 3/60) and blindness (Snellen visual acuity of < 3/60 or central visual field < 10°). The burden of DR‐related vision impairment, that is, prevalence and years lived with disability (YLD), was analyzed by sex, age, location, and sociodemographic index (SDI). A Bayesian age‐period‐cohort analysis was employed to forecast the future burden up to 2035.

**Results:**

From 1990 to 2021, the global prevalence rate and YLD rate of DR‐related vision impairment increased significantly. In 2021, 2.85 million prevalent cases and 250 117 YLDs were reported, representing 2.8‐fold and 3.0‐fold increases compared to 1990, respectively. South Asia and China were identified as the most severely burdened region and country in 2021, respectively. Throughout 1990–2021, females consistently bore a greater burden than males. In terms of SDI, the burden was predominantly concentrated in middle‐SDI countries. Predictive analysis suggests a continued increase in the number of patients and YLDs by 2035.

**Conclusions:**

Globally, there has been a substantial increase in the burden of DR‐related vision impairment among working‐age individuals, with disparities observed in terms of sex, location, and SDI. Given the projected worsening of this burden, targeted interventions are needed to address this global health challenge.

## Graphical abstract

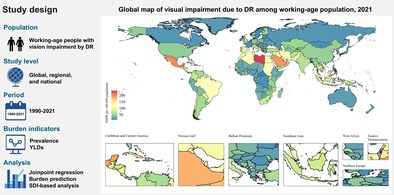

From 1990 to 2021, there has been a substantial increase in the global burden of vision impairment due to diabetic retinopathy among working‐age individuals.

**Summary**

- Burden of DR‐related vision loss increased among the working‐age population since 1990, with South Asia being the region with the highest disease burden in 2021.
- The vision impairment burden is most concentrated in middle‐SDI countries.
- By 2035, the number of vision impairment cases due to DR will further increase.

## 1 Introduction

Diabetes mellitus (DM) encompasses a spectrum of chronic metabolic disorders marked by elevated blood glucose levels [1]. Over the past few decades, the global prevalence of DM has increased dramatically, rendering it a significant public health issue [2]. In individuals with DM, persistently high blood glucose levels may lead to widespread vascular damage, causing various microvascular complications such as diabetic neuropathy, end‐stage renal disease, and diabetic retinopathy (DR) [3].

Currently, DR is one of the leading causes of vision impairment on a global scale [4]. Vision impairment has enduring impacts on individuals, families, and society. The impact is especially evident for the working‐age population (20–65 years), as vision impairment not only affects their mental health and social performance, but also hinders their employment opportunities and productivity, thereby increasing the risk of poverty [5, 6]. Improving eye health in the working‐age population may contribute to achieving the Sustainable Development Goals of the United Nations [7]. However, in many blindness prevention programs, elderly individuals and children are often the greater priorities, whereas working‐age individuals have received less attention [8].

The Global Burden of Disease Study (GBD) is the largest and most comprehensive global epidemiological survey conducted to date, providing high‐quality data on the burden of over 300 diseases and injuries across 204 countries and territories worldwide from 1990 to 2021, including DR [9]. Studies based on GBD data have investigated the burden of DR‐related vision impairment at all ages and offered useful information for public health policymaking [9, 10]. However, despite the significant socioeconomic impact of vision impairment on the working‐age population, the long‐term global trends of vision impairment due to DR, to our knowledge, have not been previously reported in this population.

This study provides a comprehensive assessment of the global, regional, and national patterns of DR‐related vision impairment in the working‐age population between 1990 and 2021, employing up‐to‐date data from GBD 2021. The aim of this analysis is to inform novel strategies aimed at reducing the vision hazards associated with DR among working‐age individuals.

## 2 Methods

### 2.1 Overview

This study is a population‐based analysis based on the GBD 2021. The GBD 2021 represents a meticulous and scientific endeavor that quantified the health impact of 371 diseases and injuries across 204 countries and territories, spanning from 1990 to 2021, categorized by sex, age, and geographical location [9]. In short, the GBD 2021 Collaborators utilized a variety of data sources as input data, including representative population‐based studies, disease registries, and healthcare utilization records [9, 11]. A series of advanced analytical procedures, such as the DisMod‐MR 2.1 model and Out‐of‐Dismod crosswalks, were employed to standardize and model the data, thereby ensuring the reliability of the output data. More details on the methodology of GBD 2021 can be found elsewhere [12].

The GBD 2021 has been approved by the Institutional Review Board Committee of the University of Washington (approval No. STUDY00009060). Since all the data utilized in this study were deidentified and publicly accessible, no additional ethics approval was needed. The study adhered to the Guidelines for Accurate and Transparent Health Estimates Reporting: the GATHER statement, and complied with the principles of the Helsinki Declaration [13].

### 2.2 Study Population

In GBD 2021, DM corresponded to codes E08‐E08.11, E08.3‐E08.9, E10‐E10.11, E10.3‐E11.1, E11.3‐E12.1, E12.3‐E13.11, E13.3‐E14.1, E14.3‐E14.9, and R73‐R73.9 in the International Classification of Diseases (ICD)‐10 and codes 249–249.31, 249.5–250.39, 250.5–250.99, 362.0–362.07, 790.2–790.29, V18‐V18.0, V42.83, V45.85, V58.67, and V77.1 in the ICD‐9 [9]. DR was defined as DM‐related damage to the retina caused by damaged blood vessels that can leak blood into the retina and cause scarring of the retina [9]. Vision impairment due to DR included low vision due to DR, which was defined as presenting visual acuity of < 6/18 to ≥ 3/60 in the better eye via the Snellen chart, and blindness due to DR, which was defined as presenting Snellen visual acuity of < 3/60 or visual field of < 10 around the central fixation point in the better eye [9]. In this study, “working‐age” was defined as 20–65 years of age [14, 15].

We employed the GBD Results Tool, available via the Global Health Data Exchange website (https://ghdx.healthdata.org/), to obtain health estimates for vision impairment associated with DR. DR data were gathered based on burden indicators (prevalence and years lived with disability [YLDs]), sex (female, male, and both), and age (in five‐year intervals for the age range of 20 to 65 years) at the global, regional (21 GBD regions), and national (204 countries and territories) levels. A single YLD signifies the loss of one entire year of healthy life as a consequence of disability or poor health conditions. For nonfatal conditions such as DR, YLDs are equivalent to disability‐adjusted life years. Data for DR‐associated vision impairment were reported in numbers and rates, accompanied by corresponding 95% uncertainty intervals (UIs).

Additionally, we obtained sociodemographic index (SDI) data for the 204 countries and territories. The SDI is a composite indicator that integrates information on income per capita, fertility rates, and education attainment, often used to assess the development level of a location (e.g., a country or territory) [16]. The SDI ranges from 0 to 1, with a country's SDI closer to 1 indicating a higher level of development and a value closer to 0 indicating a lower level of development. The GBD 2021 divided all the 204 countries and territories into five groups from low to high according to the SDI: low, low‐middle, middle, high‐middle, and high.

### 2.3 Statistical Analysis

#### 2.3.1 Joinpoint Regression

First, to evaluate the global trends in the prevalence and YLDs, we conducted a joinpoint regression analysis, with the average annual percentage changes (AAPCs) and their corresponding 95% confidence intervals (CIs) being calculated. The AAPC is a commonly used parameter to quantify the overall trend of a disease indicator (e.g., prevalence and YLDs) over a specific time period (i.e., 1990–2021 in this study). The value is determined by computing the weighted average of the annual percentage changes in joinpoint regression. If both the AAPC value and its lower limit of 95% CI were greater than 0 (equivalent to a statistically significant *P* value), it would be considered that the prevalence/YLD rate showed a statistically significant upward trend from 1990 to 2021. Similarly, if both the AAPC value and its upper limit of 95% CI were lower than 0, it would be considered that the prevalence/YLD rate showed a statistically significant downward trend between 1990 and 2021. During analysis, the optimal number of joinpoints was determined via the Monte Carlo permutation test. We set the maximum number of potential joinpoints at 5 and the minimum at 0.

#### 2.3.2 SDI‐Based Analysis

To assess the associations between DR‐related vision impairment and sociodemographic development levels, we performed an SDI‐based analysis. First, we analyzed the trends in the burden of vision impairment across the five SDI groups from 1990 to 2021. Then, at the national level, we performed a locally weighted linear scatterplot smoothing analysis to examine the relationship between the disease burden and the SDI values for each country/territory in 2021.

#### 2.3.3 Burden Prediction

Finally, we conducted a predictive analysis to predict the burden of vision impairment due to DR from 2022 to 2035. In this study, the Bayesian age‐period‐cohort (BAPC) model was used to forecast future disease burden because it is capable of processing complex, high‐dimensional, and sparse data often encountered in large‐scale epidemiological analyses such as the GBD 2021 [17]. The BAPC model, which is based on the classic age‐period‐cohort model, is a mainstream method for forecasting trends in future disease burden [16]. This model posits that age, period, and cohort effects exhibit analogous influences across contiguous time spans [18]. Through Bayesian inference, the BAPC model integrates observed data with prior distributions, thereby enabling more precise forecasts of future disease burden [18]. Integrated nested Laplace approximations (INLA) was used for Bayesian inference.

All the statistical analyses and visualizations for this study were completed using R (version 4.3.3) with the INLA package, as well as GraphPad Prism (version 9.5.1).

## 3 Results

### 3.1 Global Trends

During the study period, the global burden of DR‐related vision impairment increased substantially. Specifically, the age‐standardized prevalence rate (ASPR) increased from 42.67 per 100 000 population (95% UI, 29.66–59.24) in 1990 to 60.03 per 100 000 population (95% UI, 42.82–81.84) in 2021, whereas the age‐standardized YLD rate (ASYR) increased from 3.49 per 100 000 population (95% UI, 2.15–5.32) to 5.27 per 100 000 population (95% UI, 3.31–7.96). Correspondingly, in 2021, the numbers of prevalent cases and the YLDs also reached 2 853 377 (95% UI, 2036557–3 887 445) and 250 117 (95% UI, 157184 to 377 334), respectively, which were 2.8 times and 3.0 times greater than the numbers in 1990 (Table 1).

**TABLE 1:** Global and stratified burden of vision impairment due to diabetic retinopathy in working‐age population, 1990–2021.

	Prevalence					YLDs				
	Number, 1990	ASPR per 100 000, 1990	Number, 2021	ASPR per 100 000, 2021	AAPC, 1990–2021	Number, 1990	ASYR per 100 000, 1990	Number, 2021	ASYR per 100 000, 2021	AAPC, 1990–2021
Worldwide	1 015 893 (704 863 to 1 412 574)	42.67 (29.66 to 59.24)	2 853 377 (2 036 557 to 3 887 445)	60.03 (42.82 to 81.84)	1.13 (1.08 to 1.17)	83 489 (51 259 to 127 481)	3.49 (2.15 to 5.32)	250 117 (157 184 to 377 334)	5.27 (3.31 to 7.96)	1.35 (1.29 to 1.41)
Sex										
Female	544 394 (377 687 to 757 369)	45.83 (31.85 to 63.67)	1 590 857 (1 141 789 to 2 162 114)	66.24 (47.5 to 90.1)	1.21 (1.17 to 1.25)	44 520 (27 242 to 68 147)	3.74 (2.29 to 5.71)	141 430 (88 923 to 213 976)	5.91 (3.71 to 8.95)	1.49 (1.42 to 1.57)
Male	471 499 (327 243 to 655 946)	39.53 (27.49 to 54.92)	1 262 520 (897 368 to 1 726 421)	53.70 (38.15 to 73.46)	1.00 (0.95 to 1.05)	38 969 (23 816 to 59 470)	3.25 (1.99 to 4.95)	108 687 (68 245 to 164 290)	4.63 (2.9 to 7)	1.15 (1.1 to 1.21)
Diabetes mellitus type										
Type 1 diabetes mellitus	53 035 (33 803 to 80 305)	2.15 (1.38 to 3.24)	90 030 (59 626 to 133 887)	1.92 (1.27 to 2.87)	−0.38 (−0.44 to −0.33)	4599 (2574 to 7610)	0.19 (0.1 to 0.31)	8451 (4845 to 13 855)	0.18 (0.1 to 0.3)	−0.12 (−0.23 to −0.01)
Type 2 diabetes mellitus	962 858 (668 422 to 1 337 954)	40.52 (28.18 to 56.22)	2 763 348 (1 972 240 to 3 757 959)	58.11 (41.45 to 79.07)	1.19 (1.15 to 1.22)	78 890 (48 494 to 120 563)	3.31 (2.04 to 5.05)	241 667 (151 854 to 363 987)	5.09 (3.19 to 7.67)	1.42 (1.36 to 1.48)
Region										
Andean Latin America	3467 (2133 to 5274)	26.82 (16.67 to 40.5)	11 771 (7394 to 17 568)	34.29 (21.62 to 51.05)	0.81 (0.77 to 0.84)	201 (100 to 348)	1.55 (0.78 to 2.67)	669 (345 to 1133)	1.95 (1.01 to 3.29)	0.75 (0.68 to 0.81)
Australasia	1872 (1113 to 2885)	16.11 (9.6 to 24.78)	5057 (3090 to 7718)	23.08 (13.91 to 35.51)	1.12 (1.02 to 1.23)	104 (52 to 179)	0.9 (0.45 to 1.54)	277 (139 to 476)	1.26 (0.63 to 2.18)	1.09 (0.99 to 1.19)
Caribbean	8518 (5940 to 11 784)	57.97 (40.57 to 79.93)	24 464 (17 516 to 33 098)	83.79 (59.9 to 113.51)	1.20 (1.17 to 1.23)	856 (488 to 1417)	5.82 (3.33 to 9.61)	2734 (1573 to 4471)	9.36 (5.37 to 15.34)	1.53 (1.4 to 1.66)
Central Asia	9396 (5786 to 14 206)	31.48 (19.41 to 47.48)	26 413 (16 579 to 39 447)	47.40 (29.74 to 70.81)	1.32 (1.24 to 1.41)	522 (273 to 897)	1.75 (0.92 to 3.00)	1417 (759 to 2382)	2.55 (1.36 to 4.28)	1.20 (1.09 to 1.3)
Central Europe	17 866 (11 847 to 26 147)	21.45 (14.19 to 31.44)	29 315 (20 301 to 41 240)	31.8 (21.97 to 44.8)	1.27 (1.17 to 1.38)	1379 (817 to 2180)	1.68 (0.99 to 2.66)	2690 (1593 to 4204)	2.97 (1.75 to 4.67)	1.86 (1.76 to 1.97)
Central Latin America	64 320 (46 540 to 87 268)	118.33 (86.07 to 159.89)	205 875 (150 000 to 278 623)	140.29 (102.22 to 189.88)	0.57 (0.45 to 0.68)	6252 (3882 to 9584)	11.45 (7.17 to 17.46)	19 453 (12 354 to 29 306)	13.25 (8.42 to 19.96)	0.49 (0.36 to 0.61)
Central Sub‐Saharan Africa	2396 (1458 to 3666)	15.13 (9.28 to 23.03)	8094 (4902 to 12 350)	19.55 (11.95 to 29.67)	0.84 (0.79 to 0.89)	116 (60 to 198)	0.72 (0.37 to 1.23)	394 (199 to 676)	0.93 (0.48 to 1.59)	0.84 (0.79 to 0.88)
East Asia	211 102 (141 670 to 303 479)	37.76 (25.37 to 54.25)	572 421 (394 457 to 803 678)	47.01 (32.38 to 66.04)	0.76 (0.54 to 0.97)	13 984 (8301 to 21 813)	2.46 (1.47 to 3.83)	40 190 (24 263 to 61 968)	3.37 (2.03 to 5.22)	1.03 (0.69 to 1.36)
Eastern Europe	30 455 (19 031 to 46 207)	18.93 (11.78 to 28.75)	37 206 (23 370 to 55 909)	21.68 (13.52 to 32.71)	0.43 (0.36 to 0.51)	1794 (965 to 2977)	1.14 (0.61 to 1.89)	2058 (1126 to 3399)	1.22 (0.66 to 2.03)	0.25 (0.17 to 0.34)
Eastern Sub‐Saharan Africa	17 068 (11 450 to 23 983)	33.06 (22.32 to 46.25)	51 513 (34 502 to 72 948)	41.47 (27.99 to 58.43)	0.74 (0.68 to 0.8)	1348 (785 to 2103)	2.56 (1.5 to 3.97)	3996 (2353 to 6178)	3.13 (1.87 to 4.82)	0.67 (0.59 to 0.75)
High‐income Asia Pacific	29 110 (19 404 to 41 567)	25.29 (16.8 to 36.22)	41 196 (27 500 to 58 924)	27.75 (18.31 to 40.04)	0.31 (0.27 to 0.36)	2358 (1365 to 3725)	2.05 (1.18 to 3.24)	3121 (1795 to 5039)	2.10 (1.2 to 3.41)	0.11 (0.02 to 0.2)
High‐income North America	47 715 (31 407 to 68 219)	29.80 (19.68 to 42.48)	118 178 (84 058 to 161 747)	43.03 (30.25 to 59.34)	1.22 (1.01 to 1.44)	3819 (2226 to 5964)	2.39 (1.39 to 3.73)	11 161 (6832 to 16 870)	4.02 (2.44 to 6.11)	1.73 (1.49 to 1.98)
North Africa and Middle East	82 344 (53 622 to 119 152)	72.52 (47.52 to 104.37)	342 878 (230 832 to 489 965)	110.72 (74.81 to 157.95)	1.38 (1.35 to 1.41)	5994 (3368 to 9743)	5.21 (2.96 to 8.43)	25 004 (14 690 to 40 322)	7.95 (4.71 to 12.78)	1.39 (1.34 to 1.44)
Oceania	894 (589 to 1284)	42.34 (27.92 to 60.66)	3976 (2781 to 5542)	70.91 (49.67 to 98.93)	1.66 (1.58 to 1.75)	77 (42 to 126)	3.57 (1.99 to 5.85)	400 (233 to 634)	7.03 (4.13 to 11.12)	2.20 (2.09 to 2.32)
South Asia	206 792 (146 668 to 282 339)	51.93 (36.97 to 70.78)	580 646 (420 946 to 781 570)	63.68 (46.27 to 85.61)	0.66 (0.25 to 1.08)	19 788 (12 032 to 30 441)	4.91 (3.00 to 7.52)	61 014 (37 749 to 94 141)	6.67 (4.14 to 10.28)	1.00 (0.54 to 1.45)
Southeast Asia	99 154 (67 534 to 141 556)	57.31 (39.17 to 81.56)	343 615 (248 495 to 463 810)	79.67 (57.59 to 107.56)	1.09 (0.91 to 1.26)	7836 (4690 to 12 198)	4.50 (2.70 to 6.97)	33 039 (19 991 to 50 939)	7.66 (4.63 to 11.82)	1.78 (1.56 to 2.00)
Southern Latin America	10 295 (6707 to 15 052)	40.53 (26.42 to 59.23)	26 182 (17 611 to 37 532)	60.96 (40.9 to 87.57)	1.33 (1.15 to 1.5)	688 (374 to 1117)	2.71 (1.47 to 4.4)	1926 (1090 to 3080)	4.47 (2.52 to 7.16)	1.62 (1.36 to 1.88)
Southern Sub‐Saharan Africa	7788 (5331 to 10 944)	45.42 (31.2 to 63.67)	25 137 (17 674 to 35 075)	66.66 (46.99 to 92.93)	1.27 (1.16 to 1.38)	651 (378 to 1024)	3.73 (2.18 to 5.83)	2171 (1313 to 3373)	5.69 (3.46 to 8.81)	1.39 (1.21 to 1.57)
Tropical Latin America	71 513 (52 124 to 96 425)	119.67 (87.58 to 160.74)	181 901 (133 267 to 243 677)	124.13 (90.9 to 166.4)	0.09 (−0.19 to 0.37)	7343 (4435 to 11 263)	12.26 (7.46 to 18.68)	18 268 (11 307 to 28 213)	12.47 (7.71 to 19.27)	0.04 (−0.09 to 0.18)
Western Europe	84 496 (57 931 to 119 285)	33.13 (22.63 to 46.91)	176 868 (126 681 to 240 499)	51.62 (36.66 to 70.67)	1.46 (1.3 to 1.62)	7774 (4593 to 12 141)	3.05 (1.8 to 4.78)	17 601 (10 771 to 27 311)	5.12 (3.11 to 7.96)	1.71 (1.45 to 1.96)
Western Sub‐Saharan Africa	9332 (5905 to 13 865)	16.34 (10.44 to 24.09)	40 672 (25 441 to 60 921)	28.55 (18.04 to 42.42)	1.81 (1.73 to 1.89)	606 (336 to 985)	1.05 (0.58 to 1.69)	2534 (1395 to 4148)	1.74 (0.97 to 2.82)	1.65 (1.56 to 1.73)

*Note:* Within parentheses were 95% uncertainty intervals for numbers, ASPRs, and ASYRs, and 95% confidence intervals for AAPCs, respectively.

Abbreviations: AAPC, average annual percentage change; ASPR, age‐standardized prevalence rate; ASYR, age‐standardized YLD rate; YLDs, years lived with disability.

**FIGURE 1:**
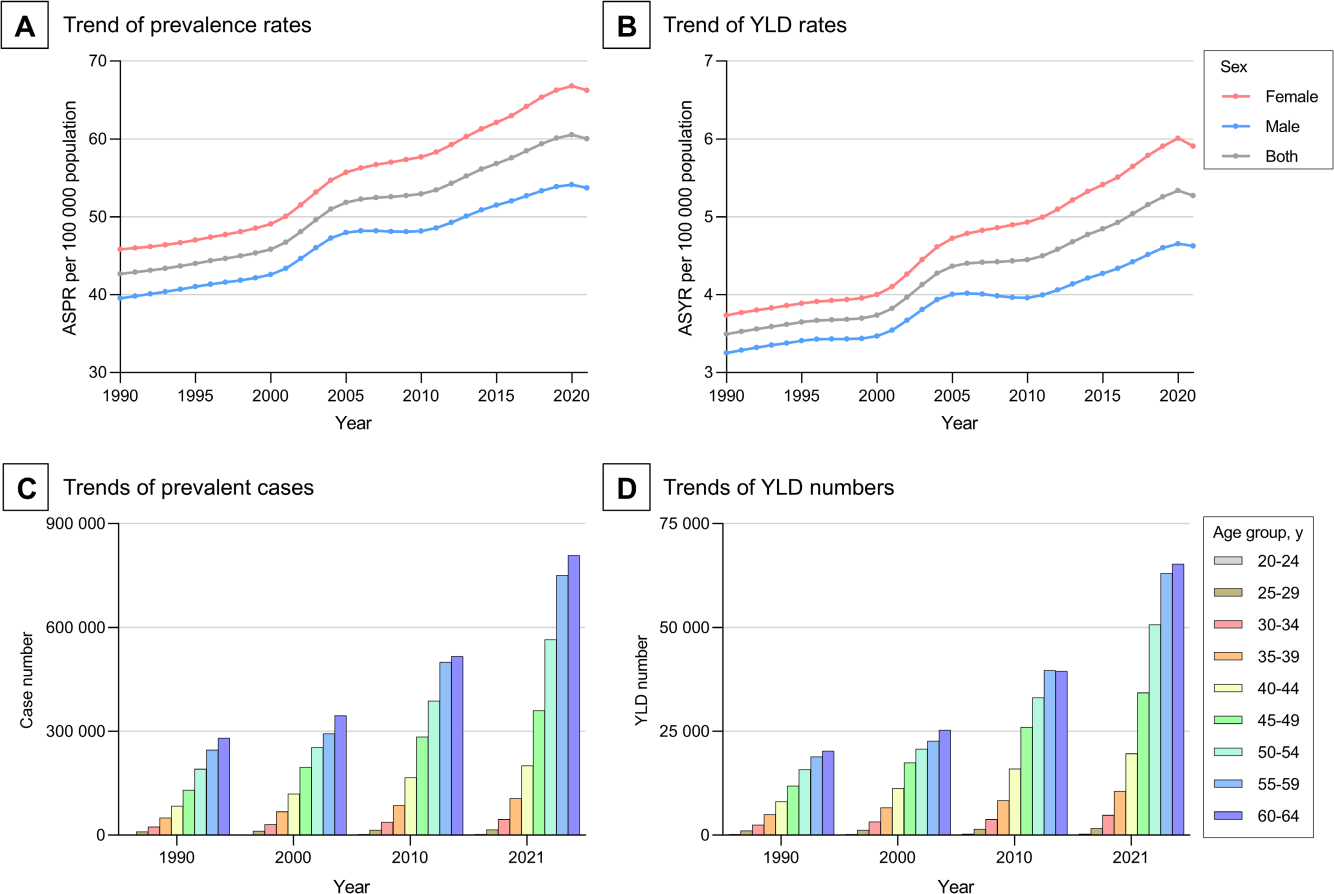
Global trends of visual impairment due to diabetic retinopathy in working‐age population, 1990–2021. ASPR, age‐standardized prevalence rate; ASYR, age‐standardized YLD rate; YLD, year lived with disability.

In terms of sex, the ASPR and ASYR increased for both females and males. Notably, females consistently had a greater disease burden than males, and the female‐to‐male ratios for ASPR (from 1.16 to 1.23) and ASYR (from 1.15 to 1.28) exhibited an increasing trend from 1990 to 2021 (Figure 1A,B, Table 1). In terms of age, during the same period, the numbers of prevalent cases and YLDs increased among all age groups, with both the largest increases observed in the 60‐to‐64‐year group. From 1990 to 2021, the numbers of prevalent cases and YLDs increased with age and both peaked in the 60‐to‐64‐year group (Figure 1C,D).

### 3.2 Regional Trends

In 2021, at the regional level, South Asia had the highest numbers of prevalent cases (580 646; 95% UI, 420946–781 570) and YLDs (61 014; 95% UI, 37749–94 141) of vision impairment due to DR among the working‐age population, whereas Central Latin America reported the highest ASPR (140.29/100000; 95% UI, 102.22–189.88) and ASYR (13.25/100000; 95% UI, 8.42–19.96). From 1990 to 2021, the fastest increase in ASPR was documented in Western Sub‐Saharan Africa (AAPC = 1.81; 95% CI, 1.73 to 1.89). However, the fastest increase in ASYR was recorded in Oceania (AAPC = 2.2; 95% CI, 2.09 to 2.32) (Table 1).

Notably, in both 1990 and 2021, the top three regions with the highest number of prevalent cases and YLDs were all located in Asia, including South Asia, East Asia, and Southeast Asia. In 2021, 52.5% of the global working‐age population affected by DR‐related vision impairment lived in these three regions, causing 53.7% of the global YLDs (Table 1).

### 3.3 National Trends

**FIGURE 2:**
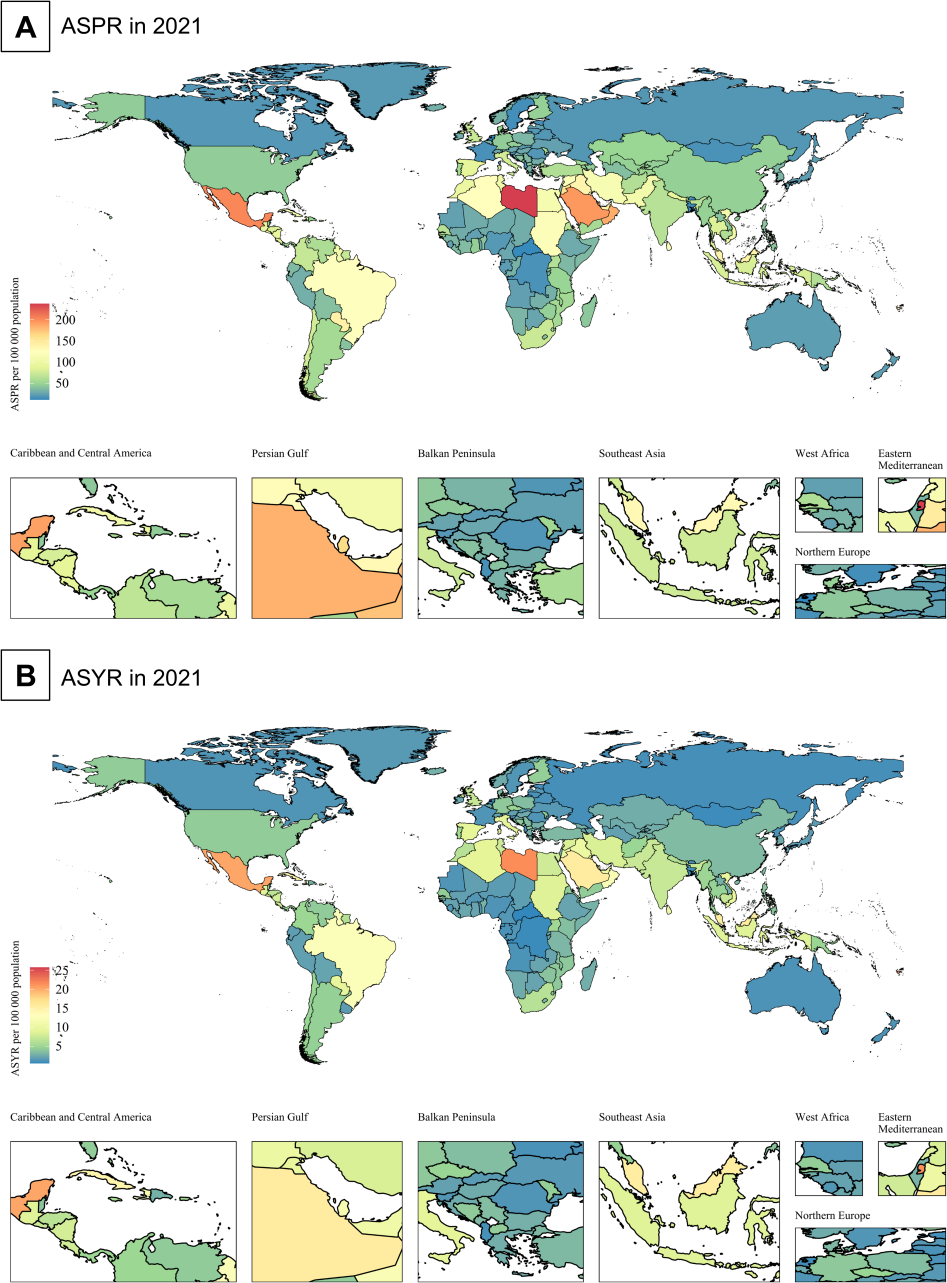
Global map of visual impairment due to diabetic retinopathy in working‐age population, 2021. ASPR, age‐standardized prevalence rate; ASYR, age‐standardized YLD rate; YLD, year lived with disability.

In 2021, at the national level, China had the highest number of DR‐related vision impairment cases among the working‐age population (558 233; 95% UI, 384418–783 319), whereas India reported the highest number of YLDs (52 677; 95% UI, 32128–82 550). In 2021, Palestine had the highest ASPR (251.91/100000; 95% UI, 174.15 to 354.10) among all countries, whereas the Netherlands had the lowest (12.06/100000; 95% UI, 7.46 to 18.76) (Figure 2, Table S1).

From 1990 to 2021, among the 204 countries (territories), 199 (97.5%) and 194 (95.1%) experienced growth in ASPR and ASYR (AAPC > 0), respectively. Among these countries, Côte d'Ivoire showed the fastest growth in ASPR (AAPC = 5.60; 95% CI, 5.22 to 5.98) and ASYR (AAPC = 5.86; 95% CI, 5.68 to 6.04), whereas Singapore had the fastest decrease in ASPR (AAPC = −0.88; 95% CI, −0.94 to −0.82) and ASYR (AAPC = −1.42; 95% CI, −1.6 to −1.24). In 2021, the ASPR of 75 countries/territories (36.8%) exceeded the global average (60.03/100000), while the ASYR of 70 countries/territories (34.3%) surpassed the global average (5.27/100000) (Figure 2, Table S1).

### 3.4 SDI‐Based Trends

**FIGURE 3:**
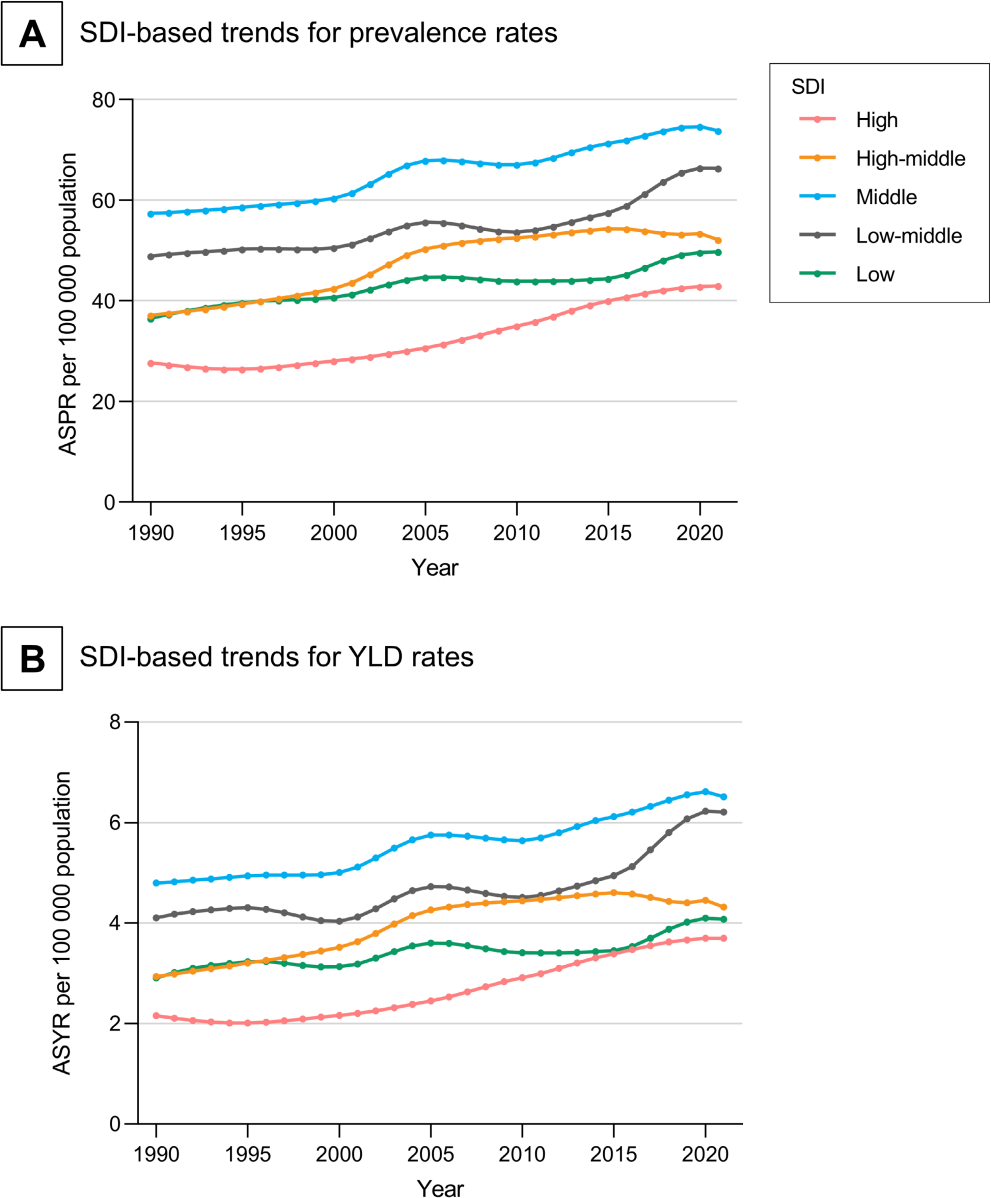
SDI‐based trends of visual impairment due to diabetic retinopathy in working‐age population, 1990–2021. ASPR, age‐standardized prevalence rate; ASYR, age‐standardized YLD rate; YLD, year lived with disability.

**FIGURE 4:**
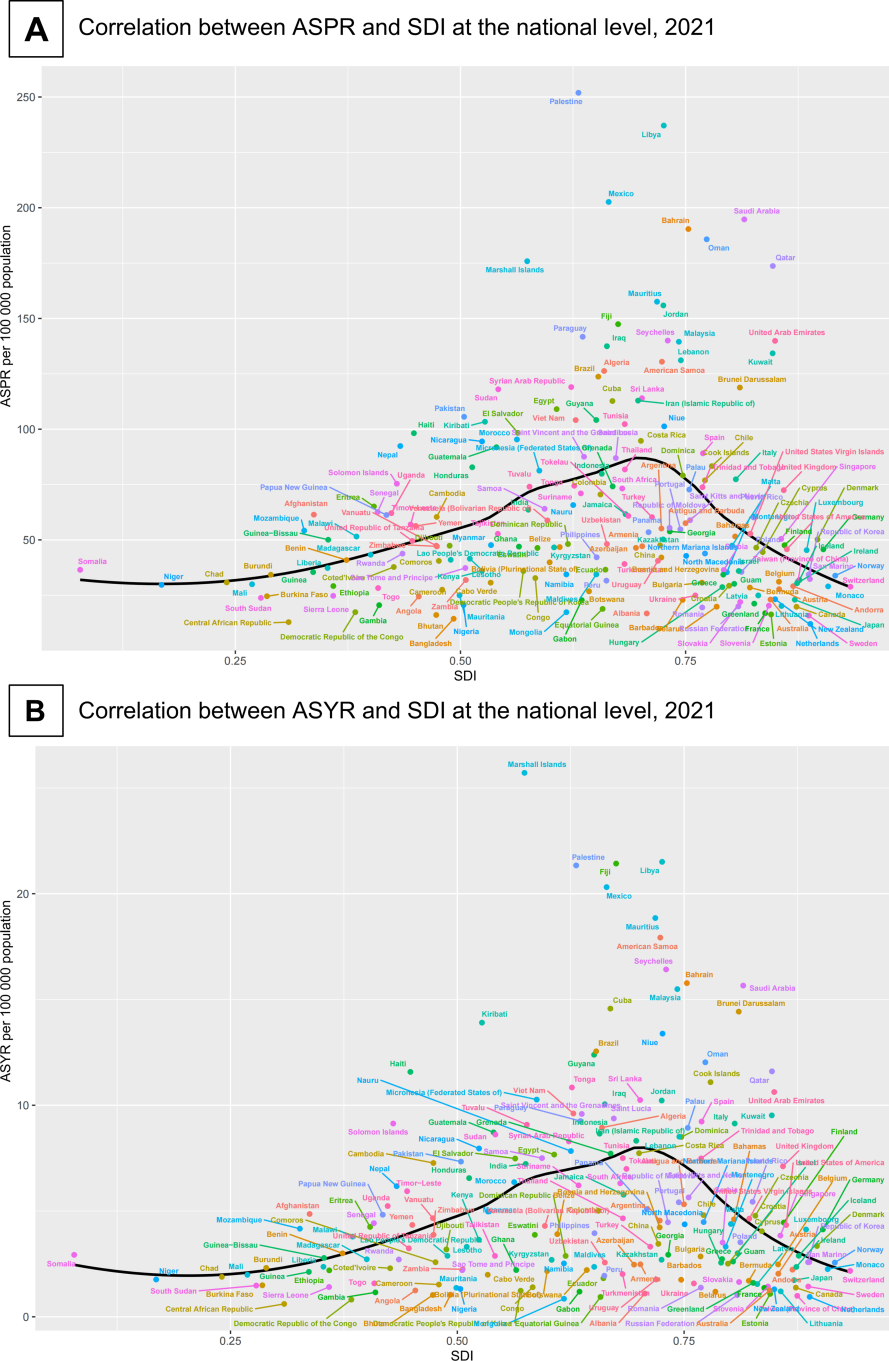
Correlation between the DR‐related vision impairment burden and SDI at the national level in working‐age population, 2021. ASPR, age‐standardized prevalence rate; ASYR, age‐standardized YLD rate; DR, diabetic retinopathy; YLD, year lived with disability.

From 1990 to 2021, the ASPR and ASYR increased across all five SDI groups. Notably, the ASPR and ASYR were consistently the highest in the middle‐SDI group, while being the lowest in the high SDI group (Figure 3). The national level analysis revealed consistent results: the ASPR and ASYR of the 204 countries exhibited an inverted V‐shaped relationship with the SDI (first increasing and then decreasing trend with increasing SDI) in 2021 (Figure 4).

### 3.5 Burden Prediction

**FIGURE 5:**
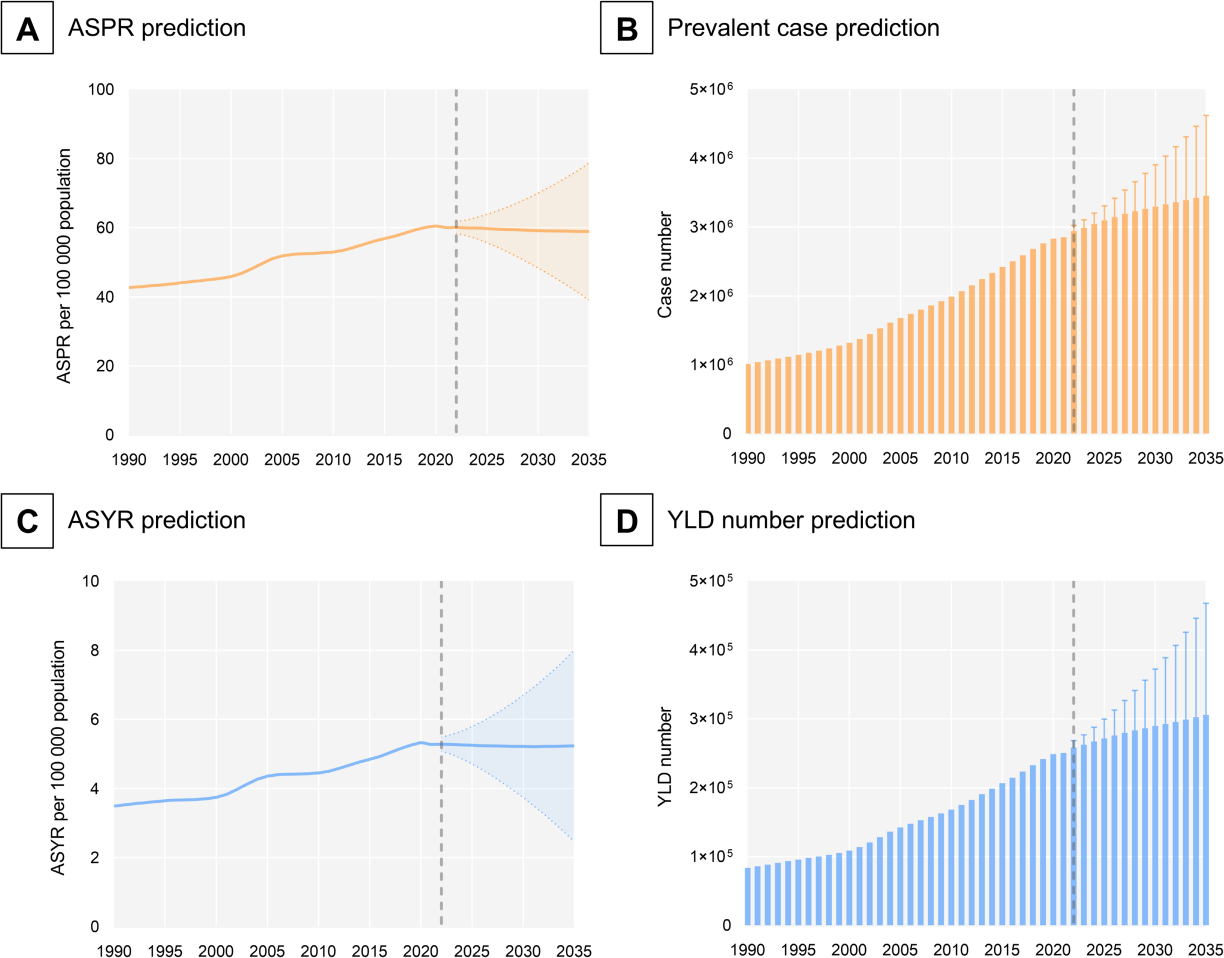
Predicted global burden of visual impairment due to diabetic retinopathy in working‐age population, 2022 to 2035. ASPR, age‐standardized prevalence rate; ASYR, age‐standardized YLD rate; YLD, year lived with disability.

The BAPC analysis suggested that the global ASPR and ASYR of DR‐related vision impairment among the working‐age population will remain relatively stable between 2022 and 2035 (Figure 5A,C). However, the global numbers of prevalent cases and YLDs are expected to increase substantially (Figure 5B,D).

## 4 Discussion

This study comprehensively evaluated the global, regional, and national patterns of DR‐related vision impairment in the working‐age population from 1990 to 2021 using updated data from GBD 2021. Our findings revealed a substantial increase in the global burden of DR‐related vision impairment throughout the study period, with notable disparities across regions and countries. We also observed an inverted V‐shaped relationship between vision impairment burden and the SDI. Furthermore, it is projected that by 2035, the number of working‐age individuals affected by DR‐related vision impairment will further increase.

In 2021, 2.85 million working‐age individuals worldwide were affected by DR‐related vision impairment, which was 2.8 times greater than the number reported in 1990. Accordingly, the YLDs in 2021 were also three times greater than those in 1990. Considering the multifaceted impacts of vision impairment on DR patients and society as a whole, this trend, given the substantial increase, is particularly alarming. This also highlights the need for targeted interventions to address the increasing burden of DR‐related vision impairment in this important population. As a chronic complication of DM, it usually takes approximately 8 years for DM patients to develop mild DR, while the progression from mild DR to more visually threatening stages (moderate, severe, or proliferative DR) only requires 1–2 years for each stage [19]. In this era, where many effective treatment options are available for DR, efficient screening procedures and prompt referrals (to facilitate timely interventions) are essential for preventing and alleviating the impacts of DR [20]. It is worth noting that adaptive optical techniques and electrophysiological techniques (such as electroretinogram) have shown promising prospects in the early (preclinical) detection of microvascular lesions and neuronal dysfunction in DR. The application of advanced technologies such as adaptive optics and electrophysiological markers may have a profound impact on future epidemiological investigations of DR and provide more high‐level evidence for the prevention and control of DR [21, 22, 23]. Furthermore, a recent study has shown that certain lipid‐lowering drug targets may reduce the risk of full‐course DR, including background DR, severe non‐proliferative DR, and proliferative DR. The development of similar drugs in the future will help reduce the burden of vision impairment related to DR [24].

We also observed sex disparities in DR‐related vision impairment among working‐age individuals. Despite the higher prevalence of DM in males than in females, the ASPR and ASYR of DR‐related vision impairment were greater for females than for males, and this sex‐related disparity continued to widen during the study period [11, 25]. On the one hand, this may be related to lower rates of vision examination and greater difficulties in accessing healthcare among females [26]. On the other hand, the inherent differences between males and females, such as pregnancy and abdominal obesity, may also be responsible [11, 27, 28].

The regional and national disparities in the vision impairment burden were striking. Regionally, South Asia had the heaviest burden of DR‐related vision impairment in working‐age individuals, followed by East Asia and Southeast Asia. In 2021, more than half of the prevalent cases worldwide lived in these regions. From this perspective, the highest burden is concentrated in Asia. Nationally, China and India had the greatest number of individuals with DR‐related vision impairment among the working‐age population. Over the past few decades, Asia has emerged as the epicenter of the global DM epidemic. It is projected that by 2025, over 60% of the world's patients with DM will be from Asia [29]. The reasons behind this phenomenon are multifaceted. First, Asia has the largest population in the world, which naturally leads to a large number of patients, especially in China and India, the two countries with the highest numbers of DM patients globally [29]. In addition, the shift in nutritional patterns, rapid urbanization, and widespread adoption of western lifestyles have collectively contributed to a sharp increase in the prevalence of DM in Asia [30]. Furthermore, from a genetic perspective, Asian populations seem to have a higher susceptibility to DM when facing adverse environmental factors than other ethnic groups [31]. Thus, with the increasing prevalence of diabetes in Asia, DR, a common complication of diabetes, has also become widespread. Although in many Asian countries (such as China, India, and Pakistan), the government has already launched national programs for the prevention and control of diabetes, the current situation suggests that further efforts are needed [31, 32, 33]. Furthermore, the rapid increase in ASPR and ASYR in Western Sub‐Saharan Africa and Oceania highlights the need for proactive actions to curb the escalating burden in these regions.

Interestingly, the SDI‐based analysis indicates that working‐age individuals in countries with middle socioeconomic development levels are more susceptible to DR‐related vision impairment than their counterparts with lower or higher levels of development. DM was once considered a “disease of affluence” and was prevalent in wealthy countries [34]. However, over the past few decades, the prevalence of DM in middle‐income countries has rapidly increased, approaching that in high‐income countries. According to the International Diabetes Federation (IDF) Diabetes Atlas 10th edition, in 2021, the prevalence of DM among adults aged 20–79 years was 11.1% in high‐income countries, 10.8% in middle‐income countries, and 5.5% in low‐income countries [35]. Despite a slightly lower prevalence, middle‐income countries often do not have as sound healthcare systems as high‐income countries, facing challenges such as limited access to healthcare services, inadequate diabetes management, and lower coverage of DR screening and treatment, which may partly explain the greater burden of DR‐related vision impairment in middle‐income countries [36, 37]. Therefore, strategies to address DR‐related vision impairment should appropriately focus on these countries.

The projection indicates that the global number of working‐age people visually impaired by DR will continue to rise between 2022 and 2035. This further emphasizes the pressing need for effective interventions for DR control, as an increase in the number of patients may exacerbate the socioeconomic consequences of vision impairment within the working‐age population, such as loss of work productivity and increased healthcare costs for society [5, 6].

Last but not least, for research based on GBD, cross‐country diagnostic variability (e.g., differences in DR screening protocols in different locations) and temporal biases in screening adoption (e.g., earlier implementation of DR screening in some high‐income countries are two points that should be noted). These factors may have, to a certain extent, undermined the accuracy and comparability of the prevalence rate and YLD estimates. For instance, high‐income countries may have more comprehensive and advanced DR screening programs. Therefore, the estimated value of the burden of DR‐related visual impairment may be closer to the actual value, which may be difficult to achieve in some low‐income countries. Therefore, in the future, it is necessary to conduct large‐scale and widespread screening of standardized diagnostic protocols to verify the findings of GBD 2021.

Our findings have several implications for public health policy and practice. First, there is a need to increase awareness and education about the risks of DR among working‐age individuals, especially those with DM. Second, eye screening should be widely promoted among high‐risk populations to achieve early detection and treatment of DR, thereby mitigating its damage to vision health. Third, targeted interventions are needed to address disparities in the burden of DR‐related vision impairment, particularly for females and individuals living in high‐burden regions (e.g., South Asia). Finally, ongoing monitoring and evaluation of DR burden are needed to guide public health decisions and track progress in reducing DR‐related vision impairment among the working‐age population.

This study has several limitations. First, although GBD 2021 employed a series of sophisticated algorithms to assess the DR burden, the quality of the raw data from various countries is uneven, which may introduce bias. Second, in some developing countries with limited medical resources (e.g., a shortage of specialized ophthalmologists), the potential underdiagnosis, misdiagnosis, and under‐registration of DR could lead to an underestimation of the overall burden. Third, since the GBD 2021 only provided data up to 2021, we were unable to analyze more recent trends in the DR burden. Despite these limitations, our findings provide useful information for the development of public health strategies for DR.

## 5 Conclusion

In conclusion, our study revealed a substantial increase in the global burden of DR‐related vision impairment among the working‐age population from 1990 to 2021. The vision impairment burden of DR differed by sex, geographical region, and sociodemographic development level. Additionally, the global number of working‐age people visually impaired by DR is projected to further increase between 2022 and 2035. These findings highlight the pressing need for proactive interventions to address the heavy burden of DR‐related vision impairment in the working‐age population.

## Author Contributions

**Yang Meng:** conceptualization, methodology, software, project administration, writing – original draft. **Yuan Liu:** conceptualization, methodology, software, project administration, writing – original draft. **Runping Duan:** resources, writing – review and editing. **Baoyi Liu:** resources, writing – review and editing. **Zhuangling Lin:** resources, writing – review and editing. **Yuan Ma:** writing – review and editing. **Lan Jiang:** writing – review and editing. **Zijian Qin:** writing – review and editing. **Tao Li:** conceptualization, methodology, resources, writing – review and editing, supervision.

## Ethics Statement

The GBD 2021 has been approved by the Institutional Review Board Committee of the University of Washington (approval No. STUDY00009060).

## Consent

The authors have nothing to report.

## Conflicts of Interest

The authors declare no conflicts of interest.

## Supporting information


**Table S1.** National burden of vision impairment due to diabetic retinopathy in working‐age population, 1990–2021.

## Data Availability

Data that support the findings of this study are available at the Global Health Data Exchange website (https://ghdx.healthdata.org/).
