# Supplementary material for: Global, Regional, and National Epidemiology of Vision Impairment due to Diabetic Retinopathy Among Working‐Age Population, 1990–2021
Source: J Diabetes. 2025 Jul 14;17(7):e70121. doi: 10.1111/1753-0407.70121 (PMC12259346; doi:10.1111/1753-0407.70121)
Supplement: Supplementary file 1 — Table S1. National burden of vision impairment due to diabetic retinopathy in working‐age population, 1990–2021. [file JDB-17-e70121-s001.pdf]

Table S1. National burden of vision impairment due to diabetic retinopathy in working-age population, 1990-2021

Location	Prevalence					YLDs				
	**Number, 1990**	**ASPR per 100000, 1990**	**Number, 2021**	**ASPR per 100000, 2021**	**AAPC, 1990-2021**	**Number, 1990**	**ASYR per 100000, 1990**	**Number, 2021**	**ASYR per 100000, 2021**	**AAPC, 1990-2021**
Afghanistan	1600 (990 to 2420)	36.79 (22.62 to 55.91)	4363 (2796 to 6508)	61.4 (39.83 to 91.21)	1.65 (1.6 to 1.7)	116 (51 to 216)	2.71 (1.18 to 5.03)	365 (175 to 648)	4.86 (2.44 to 8.47)	1.9 (1.77 to 2.03)
Albania	162 (94 to 258)	12.15 (7.08 to 19.23)	365 (224 to 561)	16.78 (10.19 to 25.89)	1.05 (0.95 to 1.16)	10 (5 to 17)	0.72 (0.36 to 1.26)	23 (12 to 39)	1.1 (0.56 to 1.83)	1.39 (1.22 to 1.55)
Algeria	6590 (4225 to 9715)	86.13 (55.64 to 126.26)	29145 (19385 to 41843)	126.27 (84.23 to 180.88)	1.22 (1.13 to 1.31)	473 (235 to 826)	6.12 (3.07 to 10.64)	2084 (1123 to 3509)	8.94 (4.87 to 14.97)	1.22 (1.14 to 1.31)
American Samoa	11 (7 to 15)	65.45 (45.95 to 90.2)	41 (31 to 55)	130.46 (96.84 to 173.86)	2.26 (2.18 to 2.35)	1 (1 to 2)	7.93 (4.08 to 13.72)	6 (3 to 9)	17.93 (10.01 to 29.07)	2.68 (2.55 to 2.82)
Andorra	5 (3 to 9)	16.56 (9.74 to 26.07)	23 (15 to 34)	29.1 (18.91 to 42.8)	1.84 (1.79 to 1.89)	0 (0 to 1)	1.1 (0.54 to 1.96)	2 (1 to 3)	2.23 (1.12 to 3.9)	2.3 (2.21 to 2.4)
Angola	631 (389 to 966)	22.21 (13.8 to 33.8)	2162 (1292 to 3349)	24.44 (14.75 to 37.65)	0.31 (0.25 to 0.37)	34 (17 to 60)	1.17 (0.58 to 2.04)	113 (56 to 197)	1.25 (0.62 to 2.16)	0.19 (0.09 to 0.29)
Antigua and Barbuda	10 (6 to 14)	40.1 (26.63 to 57.04)	37 (26 to 52)	57.55 (40.19 to 79.68)	1.18 (1.14 to 1.22)	1 (0 to 1)	3.11 (1.59 to 5.38)	3 (2 to 6)	5.32 (2.82 to 9.11)	1.76 (1.61 to 1.91)
Argentina	6448 (4232 to 9403)	36.83 (24.14 to 53.76)	15113 (10349 to 21443)	55.13 (37.72 to 78.3)	1.32 (1.23 to 1.4)	469 (239 to 802)	2.68 (1.36 to 4.58)	1248 (658 to 2079)	4.55 (2.4 to 7.58)	1.73 (1.59 to 1.86)
Armenia	650 (393 to 987)	34.39 (20.82 to 52.26)	1070 (667 to 1592)	47.14 (29.1 to 70.41)	1 (0.97 to 1.02)	36 (16 to 66)	1.93 (0.88 to 3.49)	56 (27 to 98)	2.48 (1.17 to 4.38)	0.78 (0.71 to 0.84)
Australia	1532 (902 to 2368)	15.76 (9.3 to 24.32)	4213 (2547 to 6473)	23.06 (13.74 to 35.72)	1.21 (1.1 to 1.31)	84 (40 to 146)	0.86 (0.41 to 1.5)	229 (111 to 399)	1.25 (0.6 to 2.19)	1.19 (1.1 to 1.28)
Austria	813 (486 to 1278)	16.02 (9.5 to 25.27)	2025 (1312 to 2963)	27.71 (17.71 to 41)	1.78 (1.71 to 1.86)	55 (26 to 97)	1.08 (0.51 to 1.9)	150 (79 to 262)	2.04 (1.07 to 3.55)	2.08 (1.98 to 2.18)
Azerbaijan	1045 (634 to 1592)	30.82 (18.71 to 46.83)	3502 (2169 to 5304)	46.4 (28.62 to 70.43)	1.32 (1.29 to 1.36)	58 (27 to 105)	1.71 (0.79 to 3.09)	185 (88 to 327)	2.47 (1.17 to 4.36)	1.19 (1.11 to 1.27)
Bahamas	33 (22 to 49)	34.34 (22.46 to 50.27)	137 (94 to 191)	51.55 (35.42 to 72.1)	1.32 (1.28 to 1.35)	2 (1 to 4)	2.48 (1.28 to 4.21)	12 (6 to 21)	4.61 (2.36 to 7.94)	2.02 (1.87 to 2.16)
Bahrain	197 (130 to 288)	127.53 (85.61 to 183.27)	1697 (1177 to 2412)	190.37 (132.29 to 270.22)	1.3 (1.29 to 1.31)	16 (8 to 28)	9.98 (5.35 to 16.9)	144 (82 to 237)	15.78 (9.21 to 25.56)	1.5 (1.44 to 1.57)
Bangladesh	3542 (2155 to 5389)	11.8 (7.27 to 17.84)	11860 (7454 to 17707)	14.38 (9.07 to 21.43)	0.69 (0.55 to 0.82)	245 (124 to 430)	0.8 (0.41 to 1.39)	865 (452 to 1443)	1.04 (0.55 to 1.74)	0.91 (0.75 to 1.07)
Barbados	20 (13 to 29)	16.77 (10.73 to 24.57)	56 (38 to 80)	22.91 (15.33 to 32.79)	1.02 (0.96 to 1.07)	1 (1 to 2)	1.15 (0.62 to 1.9)	4 (2 to 7)	1.75 (0.92 to 2.94)	1.38 (1.33 to 1.44)
Belarus	1432 (875 to 2155)	19.38 (11.78 to 29.33)	1612 (989 to 2468)	19.84 (12.06 to 30.57)	0.07 (0.05 to 0.09)	90 (42 to 161)	1.24 (0.58 to 2.22)	94 (44 to 165)	1.19 (0.55 to 2.09)	-0.13 (-0.21 to -0.05)
Belgium	1381 (856 to 2127)	20.21 (12.42 to 31.28)	2758 (1835 to 3963)	31.1 (20.4 to 45.13)	1.4 (1.32 to 1.48)	98 (47 to 174)	1.43 (0.69 to 2.54)	221 (113 to 386)	2.48 (1.26 to 4.32)	1.78 (1.68 to 1.89)
Belize	15 (10 to 23)	28.64 (18.33 to 42.07)	95 (65 to 134)	46.74 (32.09 to 65.76)	1.58 (1.51 to 1.66)	1 (0 to 2)	1.83 (0.94 to 3.09)	8 (4 to 14)	3.91 (1.95 to 6.86)	2.49 (2.41 to 2.56)
Benin	140 (89 to 205)	11.88 (7.61 to 17.18)	1474 (926 to 2188)	40.88 (26.03 to 60.04)	4.05 (4 to 4.11)	10 (5 to 19)	0.86 (0.4 to 1.56)	111 (51 to 200)	3 (1.4 to 5.35)	4.1 (3.99 to 4.22)
Bermuda	8 (5 to 12)	22.71 (14.12 to 34.24)	17 (11 to 24)	28.48 (17.98 to 42.18)	0.74 (0.69 to 0.79)	0 (0 to 1)	1.3 (0.66 to 2.28)	1 (0 to 2)	1.7 (0.85 to 2.89)	0.88 (0.77 to 0.99)
Bhutan	25 (15 to 39)	14.44 (8.94 to 22.28)	58 (36 to 87)	16.09 (10.1 to 24.04)	0.35 (0.26 to 0.44)	2 (1 to 3)	0.93 (0.41 to 1.67)	4 (2 to 7)	1.03 (0.49 to 1.88)	0.33 (0.22 to 0.45)
Bolivia (Plurinational State of)	692 (423 to 1059)	33.17 (20.51 to 50.45)	2262 (1401 to 3412)	39.87 (24.84 to 59.94)	0.64 (0.49 to 0.79)	39 (19 to 69)	1.86 (0.89 to 3.3)	123 (61 to 220)	2.16 (1.07 to 3.87)	0.51 (0.35 to 0.68)
Bosnia and Herzegovina	619 (399 to 920)	21.81 (14.02 to 32.45)	1203 (825 to 1696)	42.06 (28.66 to 59.48)	2.15 (2.06 to 2.23)	46 (23 to 81)	1.63 (0.83 to 2.85)	118 (61 to 203)	4.23 (2.15 to 7.29)	3.1 (2.94 to 3.25)
Botswana	74 (47 to 110)	20.02 (12.76 to 29.47)	284 (185 to 406)	26.78 (17.55 to 38.07)	0.96 (0.87 to 1.06)	6 (3 to 10)	1.58 (0.77 to 2.76)	24 (12 to 41)	2.21 (1.09 to 3.8)	1.1 (0.89 to 1.3)

Brazil	70135 (51152 to 94639)	120.08 (87.95 to 161.41)	176893 (129736 to 237000)	123.73 (90.69 to 165.88)	0.07 (-0.22 to 0.36)	7250 (4372 to 11137)	12.39 (7.52 to 18.9)	17941 (11085 to 27708)	12.55 (7.75 to 19.4)	0.03 (-0.11 to 0.17)
Brunei Darussalam	119 (86 to 162)	134.72 (99.31 to 179.23)	325 (239 to 436)	118.8 (87.55 to 158.81)	-0.4 (-0.51 to -0.3)	16 (8 to 26)	17.53 (9.81 to 28.42)	40 (22 to 65)	14.43 (8.03 to 23.52)	-0.62 (-0.69 to -0.55)
Bulgaria	1456 (951 to 2167)	21.65 (14.05 to 32.34)	1740 (1185 to 2500)	30.7 (20.79 to 44.29)	1.15 (1.07 to 1.24)	113 (59 to 192)	1.72 (0.9 to 2.92)	159 (79 to 274)	2.85 (1.41 to 4.94)	1.67 (1.55 to 1.79)
Burkina Faso	191 (120 to 292)	6.95 (4.4 to 10.53)	1515 (931 to 2283)	24.56 (15.25 to 36.71)	4.12 (3.89 to 4.35)	10 (5 to 18)	0.37 (0.19 to 0.66)	94 (46 to 166)	1.49 (0.73 to 2.6)	4.53 (4.24 to 4.82)
Burundi	449 (286 to 661)	30.83 (19.82 to 45.03)	1255 (799 to 1851)	34.26 (22.07 to 50.1)	0.34 (0.32 to 0.36)	32 (15 to 57)	2.17 (1 to 3.83)	86 (40 to 152)	2.31 (1.08 to 4.07)	0.22 (0.16 to 0.28)
Cabo Verde	24 (15 to 36)	21.1 (13.12 to 31.47)	90 (56 to 134)	30.68 (19.24 to 45.52)	1.22 (1.18 to 1.26)	2 (1 to 3)	1.33 (0.63 to 2.36)	6 (3 to 10)	1.99 (0.98 to 3.45)	1.29 (1.22 to 1.37)
Cambodia	610 (393 to 896)	19.97 (12.98 to 29.19)	5053 (3570 to 6978)	60.38 (42.78 to 83.17)	3.71 (3.51 to 3.92)	41 (22 to 71)	1.34 (0.71 to 2.3)	611 (302 to 1077)	7.27 (3.62 to 12.77)	5.74 (5.43 to 6.05)
Cameroon	578 (355 to 877)	18.84 (11.7 to 28.38)	2522 (1556 to 3809)	27.5 (17.25 to 41.11)	1.25 (1.14 to 1.35)	30 (14 to 54)	0.98 (0.47 to 1.73)	144 (72 to 251)	1.53 (0.78 to 2.66)	1.45 (1.35 to 1.56)
Canada	2023 (1173 to 3206)	12.52 (7.3 to 19.8)	5791 (3670 to 8623)	19.79 (12.31 to 29.8)	1.48 (1.44 to 1.52)	133 (65 to 237)	0.83 (0.41 to 1.47)	408 (206 to 706)	1.38 (0.7 to 2.39)	1.68 (1.57 to 1.79)
Central African Republic	75 (46 to 114)	9.14 (5.64 to 13.81)	228 (141 to 347)	12.86 (8.06 to 19.5)	1.12 (1.08 to 1.16)	3 (1 to 6)	0.38 (0.18 to 0.68)	11 (5 to 19)	0.61 (0.31 to 1.07)	1.51 (1.45 to 1.57)
Chad	246 (157 to 358)	15.12 (9.77 to 21.84)	1256 (790 to 1882)	30.81 (19.65 to 45.82)	2.33 (2.31 to 2.35)	15 (8 to 25)	0.92 (0.47 to 1.55)	79 (40 to 133)	1.89 (0.95 to 3.17)	2.34 (2.24 to 2.45)
Chile	3409 (2148 to 5085)	56.32 (35.78 to 83.47)	10132 (6658 to 14990)	76.9 (50.17 to 114.22)	1.04 (0.94 to 1.13)	200 (104 to 337)	3.31 (1.72 to 5.55)	637 (341 to 1066)	4.81 (2.56 to 8.06)	1.21 (1.06 to 1.36)
China	204939 (137684 to 294529)	38.09 (25.62 to 54.7)	558233 (384418 to 783319)	47.49 (32.7 to 66.69)	0.76 (0.54 to 0.99)	13772 (8168 to 21502)	2.52 (1.5 to 3.92)	39723 (23966 to 61289)	3.45 (2.07 to 5.35)	1.03 (0.69 to 1.37)
Colombia	7341 (4877 to 10577)	63.25 (42.42 to 90.5)	21514 (14509 to 30827)	70.51 (47.47 to 101.1)	0.36 (0.28 to 0.43)	526 (284 to 880)	4.52 (2.46 to 7.54)	1528 (798 to 2537)	5.01 (2.61 to 8.33)	0.35 (0.19 to 0.5)
Comoros	51 (32 to 76)	38.12 (24 to 56.96)	132 (83 to 198)	40.5 (25.6 to 60.83)	0.21 (0.19 to 0.22)	3 (2 to 6)	2.57 (1.29 to 4.35)	8 (4 to 15)	2.58 (1.29 to 4.44)	0.03 (-0.08 to 0.14)
Congo	199 (120 to 314)	28.37 (17.19 to 44.64)	656 (388 to 1025)	32.75 (19.55 to 50.98)	0.46 (0.41 to 0.52)	9 (4 to 16)	1.26 (0.64 to 2.23)	29 (14 to 50)	1.4 (0.7 to 2.42)	0.34 (0.25 to 0.44)
Cook Islands	3 (2 to 4)	36.26 (24.69 to 51.52)	11 (8 to 16)	83.29 (59.32 to 114.84)	2.73 (2.64 to 2.82)	0 (0 to 1)	3.87 (1.85 to 6.99)	1 (1 to 3)	11.09 (5.72 to 19.12)	3.47 (3.25 to 3.68)
Costa Rica	694 (462 to 1014)	65.78 (44.23 to 95.33)	2885 (2026 to 4021)	94.72 (66.48 to 132.13)	1.18 (1.12 to 1.25)	47 (24 to 80)	4.49 (2.32 to 7.58)	235 (127 to 393)	7.73 (4.15 to 12.96)	1.74 (1.62 to 1.87)
Cote d'Ivoire	211 (127 to 325)	6.83 (4.22 to 10.32)	3191 (1966 to 4847)	37.76 (23.56 to 56.85)	5.6 (5.22 to 5.98)	12 (6 to 21)	0.38 (0.19 to 0.65)	200 (98 to 347)	2.3 (1.14 to 3.98)	5.86 (5.68 to 6.04)
Croatia	752 (477 to 1121)	20.28 (12.8 to 30.33)	1035 (695 to 1504)	29.3 (19.53 to 42.7)	1.19 (1.16 to 1.22)	54 (26 to 93)	1.48 (0.72 to 2.55)	86 (45 to 149)	2.51 (1.28 to 4.36)	1.72 (1.61 to 1.82)
Cuba	4322 (3009 to 6005)	79.1 (55.2 to 109.63)	10845 (7784 to 14745)	112.71 (80.35 to 154.1)	1.14 (1.08 to 1.19)	508 (264 to 902)	9.27 (4.85 to 16.41)	1396 (758 to 2365)	14.57 (7.78 to 24.91)	1.46 (1.31 to 1.6)
Cyprus	181 (123 to 254)	42.86 (29.03 to 60.06)	435 (299 to 614)	44.48 (30.47 to 62.83)	0.1 (0.02 to 0.19)	17 (9 to 29)	4.11 (2.05 to 6.92)	40 (21 to 66)	4.05 (2.1 to 6.8)	-0.05 (-0.22 to 0.13)
Czechia	1879 (1246 to 2746)	27.51 (18.22 to 40.3)	3833 (2658 to 5336)	46.51 (32.1 to 64.97)	1.71 (1.62 to 1.79)	161 (82 to 281)	2.37 (1.19 to 4.15)	389 (191 to 677)	4.78 (2.33 to 8.36)	2.27 (2.12 to 2.43)
Democratic People's Republic of Korea	3162 (1953 to 4833)	28.66 (17.75 to 43.74)	7266 (4593 to 10965)	35.92 (22.69 to 54.31)	0.72 (0.67 to 0.78)	114 (52 to 211)	1.03 (0.47 to 1.9)	249 (118 to 459)	1.23 (0.58 to 2.27)	0.56 (0.46 to 0.65)
Democratic Republic of the Congo	1399 (840 to 2147)	12.73 (7.7 to 19.4)	4813 (2884 to 7407)	17.36 (10.52 to 26.53)	1.02 (0.96 to 1.08)	65 (30 to 116)	0.58 (0.28 to 1.04)	229 (108 to 402)	0.81 (0.39 to 1.42)	1.08 (0.98 to 1.18)
Denmark	1071 (662 to 1618)	32.18 (19.82 to 48.76)	2179 (1407 to 3217)	50.16 (31.93 to 74.83)	1.43 (1.35 to 1.51)	71 (33 to 129)	2.14 (0.98 to 3.86)	147 (73 to 257)	3.35 (1.64 to 5.91)	1.47 (1.37 to 1.58)

Djibouti	46 (29 to 68)	41.09 (26.24 to 60.18)	247 (157 to 365)	47.37 (30.36 to 69.56)	0.47 (0.44 to 0.49)	3 (2 to 6)	2.85 (1.48 to 4.87)	17 (8 to 29)	3.19 (1.58 to 5.45)	0.37 (0.25 to 0.48)
Dominica	12 (8 to 18)	43.99 (29.54 to 62.32)	37 (27 to 51)	79.08 (56.11 to 108.18)	1.92 (1.82 to 2.01)	1 (1 to 2)	3.64 (1.82 to 6.32)	4 (2 to 7)	8.5 (4.59 to 14.56)	2.78 (2.62 to 2.94)
Dominican Republic	783 (475 to 1202)	32.95 (20.26 to 50.12)	2798 (1797 to 4154)	48.12 (30.98 to 71.3)	1.25 (1.19 to 1.31)	43 (20 to 76)	1.82 (0.85 to 3.2)	167 (87 to 286)	2.88 (1.49 to 4.91)	1.52 (1.41 to 1.62)
Ecuador	853 (506 to 1321)	25.48 (15.37 to 39.02)	3398 (2088 to 5187)	36.56 (22.54 to 55.7)	1.18 (1.12 to 1.24)	41 (20 to 76)	1.24 (0.59 to 2.24)	169 (81 to 294)	1.82 (0.88 to 3.15)	1.25 (1.1 to 1.4)
Egypt	10672 (6676 to 15749)	54.15 (34.27 to 79.37)	51376 (33348 to 75580)	109.05 (71.09 to 160.02)	2.27 (2.2 to 2.35)	769 (345 to 1385)	3.84 (1.74 to 6.87)	3648 (1832 to 6284)	7.67 (3.9 to 13.13)	2.25 (2.13 to 2.37)
El Salvador	1002 (642 to 1477)	57 (36.81 to 83.53)	3168 (2174 to 4502)	98.33 (67.57 to 139.56)	1.78 (1.71 to 1.84)	59 (30 to 99)	3.33 (1.7 to 5.61)	241 (131 to 406)	7.48 (4.08 to 12.59)	2.64 (2.53 to 2.76)
Equatorial Guinea	25 (16 to 38)	19.04 (11.8 to 28.95)	69 (41 to 108)	18.84 (11.43 to 29.36)	-0.02 (-0.09 to 0.04)	1 (1 to 2)	1 (0.53 to 1.71)	4 (2 to 6)	0.94 (0.49 to 1.62)	-0.21 (-0.31 to -0.11)
Eritrea	503 (321 to 750)	53.02 (34.06 to 78.59)	1428 (917 to 2133)	65.08 (42.16 to 96.75)	0.66 (0.64 to 0.69)	34 (18 to 57)	3.5 (1.85 to 5.87)	95 (49 to 164)	4.25 (2.19 to 7.27)	0.62 (0.5 to 0.75)
Estonia	151 (93 to 229)	13.66 (8.35 to 20.72)	166 (106 to 249)	16.33 (10.27 to 24.56)	0.59 (0.54 to 0.64)	10 (5 to 19)	0.94 (0.45 to 1.7)	11 (5 to 19)	1.08 (0.53 to 1.89)	0.46 (0.33 to 0.59)
Eswatini	68 (43 to 100)	34.18 (22.11 to 49.98)	183 (122 to 261)	46.37 (31.14 to 66.01)	0.99 (0.91 to 1.07)	5 (3 to 10)	2.69 (1.31 to 4.72)	16 (8 to 27)	3.88 (1.98 to 6.64)	1.18 (1.04 to 1.32)
Ethiopia	4456 (3058 to 6203)	30.9 (21.4 to 42.72)	9077 (5827 to 13264)	29.16 (18.99 to 42.11)	-0.18 (-0.22 to -0.14)	386 (219 to 614)	2.63 (1.49 to 4.16)	701 (385 to 1132)	2.2 (1.21 to 3.51)	-0.56 (-0.67 to -0.46)
Fiji	165 (112 to 230)	61.71 (42.39 to 85.82)	778 (563 to 1044)	147.42 (106.66 to 197.93)	2.8 (2.63 to 2.98)	20 (10 to 36)	7.37 (3.78 to 13.01)	113 (62 to 184)	21.43 (11.78 to 34.94)	3.49 (3.31 to 3.66)
Finland	1050 (700 to 1525)	31.49 (20.96 to 45.8)	1932 (1366 to 2685)	47.58 (33.3 to 66.59)	1.33 (1.19 to 1.48)	88 (45 to 146)	2.63 (1.34 to 4.4)	182 (97 to 299)	4.46 (2.35 to 7.35)	1.72 (1.57 to 1.87)
France	4141 (2369 to 6591)	11.31 (6.42 to 18.04)	8290 (5127 to 12672)	16.89 (10.26 to 26.12)	1.3 (1.21 to 1.39)	349 (140 to 669)	0.95 (0.39 to 1.81)	674 (300 to 1281)	1.37 (0.61 to 2.6)	1.22 (1.08 to 1.36)
Gabon	67 (41 to 104)	19.57 (11.91 to 30.26)	167 (102 to 257)	22.78 (13.97 to 35.03)	0.49 (0.47 to 0.52)	4 (2 to 6)	1.05 (0.54 to 1.83)	9 (5 to 15)	1.19 (0.63 to 2.03)	0.39 (0.34 to 0.44)
Gambia	28 (17 to 44)	11.99 (7.45 to 18.25)	132 (78 to 207)	20.53 (12.38 to 31.92)	1.76 (1.66 to 1.86)	2 (1 to 3)	0.7 (0.35 to 1.22)	8 (4 to 13)	1.16 (0.58 to 2.02)	1.67 (1.46 to 1.88)
Georgia	1167 (701 to 1776)	31.07 (18.54 to 47.42)	1519 (962 to 2260)	53.82 (33.74 to 80.46)	1.77 (1.72 to 1.82)	63 (30 to 115)	1.7 (0.79 to 3.1)	82 (42 to 141)	2.93 (1.48 to 5.09)	1.77 (1.68 to 1.85)
Germany	17418 (11514 to 25462)	30.13 (19.76 to 44.25)	32503 (22624 to 45627)	45.75 (31.31 to 64.96)	1.33 (1.21 to 1.46)	1437 (733 to 2444)	2.48 (1.26 to 4.24)	2972 (1538 to 5077)	4.13 (2.12 to 7.07)	1.62 (1.41 to 1.83)
Ghana	820 (496 to 1250)	19.3 (11.82 to 29.16)	5469 (3375 to 8296)	46.96 (29.3 to 70.79)	2.86 (2.71 to 3)	37 (18 to 64)	0.85 (0.44 to 1.5)	262 (133 to 453)	2.21 (1.13 to 3.8)	3.08 (2.79 to 3.36)
Greece	1597 (986 to 2448)	21.65 (13.19 to 33.46)	2846 (1886 to 4108)	34.3 (22.37 to 50.06)	1.5 (1.42 to 1.59)	119 (54 to 217)	1.61 (0.73 to 2.93)	229 (114 to 384)	2.73 (1.35 to 4.6)	1.72 (1.6 to 1.84)
Greenland	3 (2 to 5)	11.29 (6.61 to 17.85)	10 (6 to 14)	21.21 (13.54 to 31.39)	2.07 (2.01 to 2.12)	0 (0 to 0)	0.76 (0.38 to 1.32)	1 (0 to 1)	1.6 (0.79 to 2.78)	2.46 (2.33 to 2.58)
Grenada	16 (11 to 22)	49.31 (33.77 to 69.98)	52 (37 to 71)	74.15 (52.11 to 101.85)	1.34 (1.29 to 1.4)	1 (1 to 2)	4.26 (2.13 to 7.4)	5 (3 to 9)	7.72 (3.96 to 13.22)	1.94 (1.82 to 2.06)
Guam	11 (7 to 17)	19.62 (12.33 to 29.98)	35 (23 to 50)	30.16 (19.97 to 43.48)	1.39 (1.27 to 1.51)	1 (0 to 1)	1.49 (0.73 to 2.67)	3 (1 to 5)	2.61 (1.23 to 4.64)	1.81 (1.68 to 1.94)
Guatemala	1094 (708 to 1606)	45.56 (29.83 to 66.3)	6116 (4265 to 8484)	91.85 (64.36 to 126.8)	2.32 (2.23 to 2.41)	71 (36 to 121)	2.97 (1.53 to 5.04)	581 (305 to 976)	8.72 (4.62 to 14.59)	3.56 (3.47 to 3.66)
Guinea-Bissau	72 (45 to 107)	27.64 (17.37 to 40.87)	272 (166 to 414)	50.06 (31.03 to 75.37)	1.92 (1.83 to 2.02)	4 (2 to 7)	1.54 (0.73 to 2.71)	16 (7 to 28)	2.78 (1.32 to 4.97)	1.9 (1.79 to 2.01)
Guinea	296 (184 to 445)	15.22 (9.54 to 22.74)	1308 (800 to 1973)	35.44 (21.92 to 52.99)	2.75 (2.69 to 2.82)	17 (9 to 30)	0.88 (0.44 to 1.55)	80 (38 to 143)	2.12 (1.02 to 3.78)	2.87 (2.77 to 2.96)

Guyana	158 (107 to 220)	63.04 (43.21 to 87.2)	436 (313 to 597)	104.14 (74.61 to 142.69)	1.63 (1.56 to 1.69)	16 (8 to 27)	6.26 (3.27 to 10.62)	52 (28 to 88)	12.39 (6.7 to 20.9)	2.23 (2.1 to 2.36)
Haiti	1271 (870 to 1775)	57.77 (39.78 to 80.34)	5258 (3707 to 7215)	98.18 (69.62 to 133.85)	1.73 (1.7 to 1.76)	121 (62 to 209)	5.5 (2.82 to 9.48)	623 (322 to 1104)	11.58 (6.08 to 20.27)	2.44 (2.31 to 2.57)
Honduras	782 (508 to 1131)	59.1 (38.71 to 84.83)	3382 (2330 to 4791)	82.81 (57.35 to 116.61)	1.1 (1.07 to 1.13)	52 (27 to 87)	3.94 (2.02 to 6.58)	268 (143 to 449)	6.56 (3.53 to 10.92)	1.65 (1.53 to 1.77)
Hungary	1505 (977 to 2228)	20.28 (13.1 to 30.1)	2191 (1474 to 3164)	28.69 (19.2 to 41.56)	1.14 (1.07 to 1.21)	120 (54 to 219)	1.64 (0.72 to 3.01)	193 (93 to 352)	2.56 (1.22 to 4.68)	1.5 (1.36 to 1.64)
Iceland	21 (12 to 34)	15.77 (9.29 to 24.96)	79 (54 to 114)	31.88 (21.43 to 46.25)	2.3 (2.2 to 2.41)	2 (1 to 3)	1.13 (0.55 to 2.04)	7 (4 to 13)	2.89 (1.41 to 5.05)	3.09 (3.01 to 3.18)
India	160958 (114235 to 218613)	49.28 (35.09 to 66.8)	462725 (337211 to 620481)	63.63 (46.46 to 85.22)	0.83 (0.6 to 1.06)	16709 (10085 to 25820)	5.05 (3.07 to 7.79)	52677 (32128 to 82550)	7.23 (4.42 to 11.31)	1.15 (0.85 to 1.46)
Indonesia	42575 (28148 to 62477)	60.87 (40.37 to 89.06)	137890 (95654 to 193532)	79.86 (55.42 to 112.03)	0.94 (0.74 to 1.14)	3798 (2171 to 6113)	5.38 (3.09 to 8.6)	14962 (8733 to 24082)	8.66 (5.06 to 13.93)	1.62 (1.35 to 1.89)
Iran (Islamic Republic of)	16687 (10999 to 23966)	90.98 (60.26 to 130.09)	57590 (38669 to 81958)	112.89 (76.04 to 160.29)	0.69 (0.62 to 0.75)	1232 (707 to 1984)	6.69 (3.85 to 10.74)	4291 (2550 to 6766)	8.32 (4.98 to 13.07)	0.69 (0.62 to 0.76)
Iraq	5592 (3686 to 8168)	113.96 (75.76 to 165.28)	23471 (15768 to 34135)	137.46 (92.77 to 199.29)	0.6 (0.58 to 0.63)	434 (228 to 744)	8.64 (4.64 to 14.6)	1746 (948 to 2914)	10.05 (5.54 to 16.6)	0.48 (0.39 to 0.57)
Ireland	249 (145 to 395)	13.38 (7.81 to 21.23)	1086 (718 to 1577)	30.42 (19.93 to 44.54)	2.69 (2.58 to 2.8)	16 (7 to 30)	0.88 (0.4 to 1.62)	85 (43 to 146)	2.36 (1.19 to 4.07)	3.26 (3.07 to 3.45)
Israel	521 (321 to 793)	22.44 (13.91 to 34.04)	1925 (1282 to 2775)	35.95 (23.87 to 51.93)	1.49 (1.4 to 1.58)	38 (19 to 68)	1.66 (0.84 to 2.93)	161 (82 to 281)	3.01 (1.53 to 5.25)	1.94 (1.71 to 2.16)
Italy	21850 (15568 to 29334)	55.34 (39.25 to 74.54)	38813 (28913 to 50619)	77.37 (57.13 to 101.73)	1.2 (0.78 to 1.62)	2437 (1463 to 3756)	6.19 (3.7 to 9.59)	4594 (2858 to 7048)	9.14 (5.63 to 14.12)	1.3 (0.73 to 1.88)
Jamaica	292 (188 to 429)	34.13 (22.22 to 49.97)	1050 (729 to 1461)	61.63 (42.79 to 85.79)	1.95 (1.86 to 2.04)	20 (10 to 35)	2.39 (1.22 to 4.11)	98 (51 to 174)	5.77 (3 to 10.22)	2.9 (2.71 to 3.09)
Japan	20852 (13785 to 29771)	22.57 (14.82 to 32.41)	21908 (14485 to 31673)	22.98 (15 to 33.53)	0.09 (0 to 0.19)	1674 (971 to 2635)	1.81 (1.05 to 2.86)	1654 (948 to 2636)	1.73 (0.98 to 2.78)	-0.11 (-0.24 to 0.01)
Jordan	1241 (804 to 1831)	127.2 (83.11 to 187.08)	8703 (5775 to 12732)	155.88 (103.96 to 227.29)	0.64 (0.55 to 0.74)	87 (45 to 145)	8.7 (4.64 to 14.38)	581 (319 to 961)	10.22 (5.71 to 16.78)	0.53 (0.47 to 0.6)
Kazakhstan	2924 (1797 to 4433)	36.08 (22.23 to 54.6)	5814 (3630 to 8734)	50.21 (31.3 to 75.5)	1.06 (1.01 to 1.1)	162 (76 to 289)	2 (0.94 to 3.56)	312 (152 to 545)	2.7 (1.31 to 4.71)	0.94 (0.85 to 1.03)
Kenya	1372 (918 to 1944)	24.32 (16.4 to 34.31)	6137 (4287 to 8466)	36.03 (25.29 to 49.59)	1.27 (1.12 to 1.41)	124 (74 to 195)	2.14 (1.29 to 3.33)	639 (378 to 997)	3.65 (2.19 to 5.63)	1.74 (1.54 to 1.94)
Kiribati	11 (7 to 15)	42.54 (29.14 to 59.4)	56 (40 to 76)	103.35 (74.49 to 139.98)	2.92 (2.82 to 3.02)	1 (1 to 2)	4.37 (2.12 to 7.73)	8 (4 to 13)	13.91 (7.57 to 23.04)	3.85 (3.7 to 4)
Kuwait	552 (355 to 808)	102.99 (67.78 to 149.28)	3589 (2383 to 5181)	134.25 (90.23 to 193)	0.87 (0.81 to 0.93)	42 (21 to 72)	7.35 (3.91 to 12.55)	263 (142 to 442)	9.52 (5.27 to 15.71)	0.84 (0.75 to 0.93)
Kyrgyzstan	619 (381 to 928)	33.52 (20.69 to 50.21)	1587 (996 to 2380)	46.61 (29.3 to 69.79)	1.06 (0.99 to 1.12)	37 (17 to 67)	2.03 (0.91 to 3.63)	92 (42 to 165)	2.69 (1.23 to 4.82)	0.93 (0.83 to 1.03)
Lao People's Democratic Republic	372 (236 to 560)	27.52 (17.53 to 41.33)	1347 (899 to 1930)	41.01 (27.48 to 58.55)	1.3 (1.27 to 1.34)	21 (11 to 35)	1.57 (0.84 to 2.6)	95 (48 to 160)	2.88 (1.45 to 4.83)	1.97 (1.9 to 2.05)
Latvia	371 (226 to 565)	19.17 (11.61 to 29.32)	391 (243 to 587)	24.93 (15.3 to 37.64)	0.85 (0.81 to 0.89)	23 (11 to 42)	1.22 (0.57 to 2.2)	24 (12 to 42)	1.55 (0.75 to 2.74)	0.78 (0.67 to 0.89)
Lebanon	1370 (896 to 2006)	96.73 (63.26 to 141.68)	3944 (2627 to 5722)	131.11 (87.61 to 189.84)	0.98 (0.93 to 1.03)	88 (46 to 151)	6.23 (3.23 to 10.71)	258 (140 to 433)	8.51 (4.65 to 14.23)	1.02 (0.93 to 1.11)
Lesotho	145 (92 to 217)	28.62 (18.12 to 42.53)	289 (189 to 420)	41.51 (27.39 to 60.11)	1.2 (1.1 to 1.29)	11 (6 to 20)	2.19 (1.1 to 3.88)	23 (11 to 39)	3.31 (1.61 to 5.6)	1.34 (1.21 to 1.47)
Liberia	187 (116 to 278)	27.78 (17.44 to 41.01)	610 (384 to 911)	37.3 (23.85 to 55.25)	0.93 (0.82 to 1.04)	12 (6 to 21)	1.74 (0.84 to 3.03)	40 (21 to 67)	2.37 (1.23 to 3.95)	0.97 (0.78 to 1.16)
Libya	1866 (1254 to 2716)	148.88 (100.74 to 216.05)	9294 (6493 to 13045)	237.11 (166.23 to 332.52)	1.69 (1.5 to 1.88)	156 (84 to 260)	12.19 (6.66 to 20.16)	861 (488 to 1377)	21.51 (12.37 to 34.2)	2.1 (1.8 to 2.4)

Lithuania	457 (280 to 696)	18.39 (11.18 to 28.08)	482 (295 to 742)	20.16 (12.2 to 31.16)	0.3 (0.28 to 0.33)	29 (13 to 51)	1.16 (0.55 to 2.07)	28 (14 to 49)	1.2 (0.59 to 2.13)	0.12 (0.01 to 0.22)
Luxembourg	39 (23 to 61)	14.55 (8.5 to 23)	231 (160 to 323)	45.37 (31.28 to 63.9)	3.73 (3.6 to 3.87)	3 (1 to 5)	0.96 (0.44 to 1.73)	21 (11 to 35)	4.13 (2.18 to 6.88)	4.83 (4.69 to 4.98)
Madagascar	1254 (800 to 1869)	37.59 (24.12 to 55.8)	3878 (2478 to 5756)	43.32 (27.86 to 63.99)	0.48 (0.39 to 0.57)	85 (42 to 146)	2.5 (1.25 to 4.3)	249 (127 to 422)	2.72 (1.39 to 4.62)	0.3 (0.22 to 0.38)
Malawi	1133 (729 to 1642)	43.88 (28.48 to 63.3)	2669 (1735 to 3916)	51.5 (33.83 to 75.31)	0.53 (0.46 to 0.6)	89 (45 to 149)	3.34 (1.72 to 5.64)	204 (104 to 346)	3.79 (1.97 to 6.38)	0.41 (0.27 to 0.55)
Malaysia	7884 (5736 to 10577)	123.44 (90.23 to 165.25)	24544 (17881 to 33211)	139.48 (101.83 to 188.4)	0.39 (0.11 to 0.68)	800 (445 to 1315)	12.44 (7.02 to 20.27)	2733 (1493 to 4441)	15.49 (8.51 to 25.06)	0.72 (0.4 to 1.05)
Maldives	19 (12 to 28)	27.82 (17.9 to 41.08)	92 (61 to 135)	34.44 (23.06 to 50.11)	0.68 (0.63 to 0.74)	1 (1 to 2)	1.66 (0.9 to 2.79)	6 (3 to 11)	2.36 (1.21 to 4.05)	1.13 (0.99 to 1.28)
Mali	365 (226 to 544)	13.51 (8.44 to 19.99)	1847 (1124 to 2777)	29.98 (18.46 to 44.68)	2.6 (2.51 to 2.7)	24 (11 to 42)	0.88 (0.42 to 1.54)	125 (56 to 228)	1.99 (0.89 to 3.61)	2.65 (2.44 to 2.86)
Malta	83 (55 to 118)	36.51 (24.3 to 52.07)	158 (110 to 221)	47.38 (32.57 to 66.79)	0.83 (0.79 to 0.88)	7 (4 to 12)	3.24 (1.63 to 5.51)	15 (8 to 25)	4.39 (2.24 to 7.37)	0.96 (0.87 to 1.06)
Marshall Islands	8 (6 to 12)	79.68 (55.65 to 110.27)	48 (35 to 64)	175.85 (130.54 to 234.72)	2.62 (2.5 to 2.75)	1 (1 to 2)	10.14 (5.32 to 17.38)	7 (4 to 11)	25.71 (14.42 to 41.3)	3.11 (2.95 to 3.27)
Mauritania	120 (72 to 186)	20.67 (12.43 to 31.61)	345 (204 to 534)	24.98 (14.92 to 38.41)	0.6 (0.57 to 0.64)	7 (3 to 13)	1.21 (0.55 to 2.17)	19 (9 to 36)	1.37 (0.63 to 2.52)	0.38 (0.27 to 0.48)
Mauritius	342 (238 to 480)	74.29 (51.93 to 104.02)	1603 (1196 to 2129)	157.57 (117.15 to 209.77)	2.47 (2.31 to 2.63)	32 (17 to 52)	6.82 (3.63 to 11.1)	190 (109 to 302)	18.85 (10.65 to 30.24)	3.36 (3.14 to 3.58)
Mexico	49911 (36601 to 67231)	178.96 (131.95 to 240.16)	154221 (114141 to 207679)	202.62 (149.97 to 272.94)	0.41 (0.23 to 0.59)	5266 (3277 to 8097)	18.8 (11.81 to 28.73)	15471 (9907 to 23290)	20.31 (13.01 to 30.57)	0.27 (0.07 to 0.47)
Micronesia (Federated States of)	8 (5 to 11)	27.77 (18.16 to 40.03)	45 (32 to 61)	81.27 (57.99 to 110.92)	3.58 (3.24 to 3.93)	1 (0 to 1)	2.42 (1.11 to 4.34)	6 (3 to 10)	10.27 (5.4 to 17.58)	4.96 (4.57 to 5.35)
Monaco	4 (2 to 6)	15.56 (9.09 to 24.7)	10 (6 to 14)	29.01 (18.81 to 42.79)	2.04 (1.97 to 2.11)	0 (0 to 0)	1.03 (0.48 to 1.86)	1 (0 to 1)	2.25 (1.16 to 3.83)	2.57 (2.45 to 2.68)
Mongolia	91 (50 to 147)	14.39 (8.08 to 23.25)	308 (174 to 495)	17.34 (9.89 to 27.88)	0.58 (0.45 to 0.72)	5 (2 to 9)	0.76 (0.37 to 1.36)	15 (7 to 28)	0.86 (0.4 to 1.54)	0.36 (0.25 to 0.46)
Montenegro	85 (55 to 126)	22.19 (14.37 to 32.97)	172 (118 to 245)	35.38 (24.01 to 50.4)	1.51 (1.47 to 1.55)	6 (3 to 11)	1.67 (0.83 to 2.89)	16 (8 to 28)	3.32 (1.64 to 5.9)	2.22 (2.14 to 2.3)
Morocco	5251 (3459 to 7571)	59.9 (39.85 to 85.77)	20882 (13745 to 30730)	95.36 (62.76 to 140.35)	1.52 (1.5 to 1.55)	370 (179 to 651)	4.17 (2.05 to 7.29)	1394 (731 to 2343)	6.37 (3.34 to 10.7)	1.37 (1.29 to 1.46)
Mozambique	1535 (1022 to 2188)	36.76 (24.69 to 52.21)	4481 (2909 to 6497)	54.19 (35.52 to 78.02)	1.29 (1.16 to 1.42)	122 (60 to 205)	2.88 (1.43 to 4.85)	351 (173 to 590)	4.17 (2.08 to 6.98)	1.24 (1.12 to 1.36)
Myanmar	4071 (2692 to 5997)	26.89 (17.84 to 39.5)	15098 (10624 to 21078)	47.63 (33.52 to 66.52)	1.85 (1.73 to 1.96)	285 (140 to 496)	1.88 (0.92 to 3.26)	1577 (755 to 2802)	4.97 (2.38 to 8.84)	3.18 (2.92 to 3.45)
Namibia	131 (83 to 193)	31.13 (20.01 to 45.73)	323 (206 to 472)	34.41 (22.14 to 50.01)	0.3 (0.25 to 0.35)	10 (5 to 18)	2.4 (1.12 to 4.29)	24 (12 to 42)	2.52 (1.26 to 4.37)	0.13 (-0.03 to 0.29)
Nauru	1 (1 to 1)	27.97 (18.48 to 40.45)	3 (2 to 4)	65.72 (46.73 to 90.55)	2.8 (2.78 to 2.82)	0 (0 to 0)	2.43 (1.19 to 4.22)	0 (0 to 1)	7.84 (4.16 to 13.46)	3.85 (3.76 to 3.93)
Nepal	3506 (2277 to 5098)	53.61 (35.03 to 77.65)	13166 (8856 to 18862)	92.37 (62.27 to 132.14)	1.73 (1.58 to 1.88)	222 (115 to 372)	3.36 (1.76 to 5.61)	883 (495 to 1405)	6.17 (3.47 to 9.81)	1.96 (1.83 to 2.1)
Netherlands	683 (415 to 1048)	7.38 (4.49 to 11.31)	1665 (1055 to 2549)	12.06 (7.46 to 18.76)	1.6 (1.52 to 1.68)	53 (28 to 88)	0.57 (0.3 to 0.95)	131 (66 to 231)	0.94 (0.47 to 1.66)	1.65 (1.58 to 1.72)
New Zealand	339 (206 to 522)	17.86 (10.87 to 27.45)	845 (521 to 1277)	23.19 (14.08 to 35.34)	0.84 (0.77 to 0.91)	20 (10 to 35)	1.06 (0.53 to 1.83)	48 (24 to 83)	1.3 (0.66 to 2.28)	0.67 (0.61 to 0.74)
Nicaragua	685 (457 to 985)	67.75 (45.66 to 96.6)	2929 (2028 to 4093)	94.47 (65.64 to 131.51)	1.08 (1.04 to 1.13)	51 (27 to 86)	5.01 (2.64 to 8.48)	247 (132 to 413)	7.96 (4.28 to 13.21)	1.51 (1.4 to 1.61)
Niger	186 (113 to 277)	9.24 (5.77 to 13.62)	1743 (1077 to 2593)	29.7 (18.53 to 43.8)	3.83 (3.75 to 3.92)	11 (6 to 20)	0.55 (0.28 to 0.96)	105 (49 to 185)	1.77 (0.83 to 3.09)	3.81 (3.66 to 3.97)

Nigeria	4580 (2830 to 6960)	16.06 (10.02 to 24.22)	14182 (8588 to 21932)	20.61 (12.64 to 31.58)	0.79 (0.71 to 0.87)	322 (175 to 528)	1.11 (0.61 to 1.81)	926 (495 to 1538)	1.32 (0.72 to 2.17)	0.54 (0.41 to 0.67)
Niue	0 (0 to 1)	44.92 (31 to 62.6)	1 (1 to 2)	101.26 (73.71 to 136.16)	2.69 (2.6 to 2.78)	0 (0 to 0)	4.76 (2.31 to 8.26)	0 (0 to 0)	13.39 (7.27 to 22.29)	3.43 (3.3 to 3.56)
North Macedonia	295 (196 to 430)	25.06 (16.59 to 36.51)	761 (527 to 1061)	42.78 (29.53 to 59.76)	1.74 (1.72 to 1.76)	25 (12 to 42)	2.1 (1.05 to 3.62)	77 (39 to 133)	4.37 (2.21 to 7.61)	2.37 (2.29 to 2.44)
Northern Mariana Islands	3 (2 to 5)	22.44 (14.25 to 33.63)	17 (12 to 24)	43.85 (30.38 to 61.3)	2.2 (2.16 to 2.23)	0 (0 to 1)	1.83 (0.87 to 3.26)	2 (1 to 3)	4.48 (2.16 to 7.94)	2.96 (2.87 to 3.05)
Norway	655 (423 to 960)	26.12 (16.85 to 38.29)	1348 (903 to 1922)	33.84 (22.43 to 48.65)	0.82 (0.78 to 0.86)	46 (27 to 76)	1.85 (1.07 to 3.01)	103 (60 to 160)	2.56 (1.49 to 4.01)	1.05 (0.93 to 1.17)
Oman	520 (323 to 774)	95.9 (61.13 to 139.39)	3500 (2226 to 5158)	185.76 (120.92 to 269.89)	2.15 (2.01 to 2.3)	36 (17 to 64)	6.45 (3.17 to 11.24)	238 (118 to 411)	12.03 (6.3 to 20.21)	2.03 (1.91 to 2.15)
Pakistan	38761 (25717 to 56573)	112.12 (74.71 to 163.19)	92838 (61887 to 134724)	105.59 (70.84 to 152.82)	-0.19 (-0.24 to -0.14)	2610 (1415 to 4314)	7.45 (4.07 to 12.25)	6584 (3652 to 10832)	7.34 (4.12 to 11.99)	-0.05 (-0.13 to 0.03)
Palau	2 (1 to 3)	30.61 (20.45 to 44.04)	12 (9 to 16)	72.73 (52.47 to 99.21)	2.84 (2.75 to 2.93)	0 (0 to 0)	2.83 (1.33 to 4.85)	1 (1 to 2)	8.93 (4.67 to 15.14)	3.8 (3.64 to 3.96)
Palestine	964 (639 to 1409)	179.09 (119.5 to 260.77)	4770 (3284 to 6725)	251.91 (174.15 to 354.1)	1.11 (0.95 to 1.28)	79 (43 to 132)	14.58 (7.9 to 24.1)	409 (240 to 663)	21.34 (12.64 to 34.31)	1.26 (1.14 to 1.38)
Panama	288 (186 to 421)	32.43 (21.11 to 47.06)	1290 (902 to 1770)	53.47 (37.36 to 73.42)	1.63 (1.6 to 1.67)	21 (10 to 36)	2.3 (1.13 to 3.98)	123 (63 to 210)	5.09 (2.6 to 8.68)	2.6 (2.48 to 2.72)
Papua New Guinea	548 (342 to 822)	41.94 (26.36 to 62.69)	2398 (1568 to 3491)	61.24 (40.14 to 89.07)	1.21 (1.14 to 1.28)	39 (19 to 69)	2.97 (1.41 to 5.22)	193 (101 to 325)	4.83 (2.56 to 8.14)	1.57 (1.51 to 1.63)
Paraguay	1378 (912 to 1992)	102.08 (68.15 to 146.65)	5008 (3467 to 6990)	141.73 (98.57 to 197.13)	1.09 (0.97 to 1.2)	93 (50 to 157)	6.88 (3.72 to 11.56)	327 (179 to 536)	9.25 (5.08 to 15.1)	0.97 (0.83 to 1.1)
Peru	1921 (1176 to 2937)	25.64 (15.85 to 38.91)	6110 (3827 to 9086)	31.59 (19.85 to 46.85)	0.68 (0.6 to 0.76)	120 (55 to 216)	1.6 (0.73 to 2.86)	377 (177 to 670)	1.94 (0.91 to 3.45)	0.64 (0.54 to 0.75)
Philippines	7615 (5204 to 10620)	37.39 (25.67 to 51.96)	23338 (16441 to 31993)	42.14 (29.74 to 57.7)	0.38 (0.32 to 0.44)	673 (403 to 1046)	3.23 (1.96 to 5)	2204 (1342 to 3397)	3.95 (2.42 to 6.08)	0.65 (0.55 to 0.75)
Poland	5977 (4023 to 8586)	25.06 (16.85 to 36.02)	10758 (7617 to 14780)	35.55 (25.11 to 48.86)	1.11 (0.98 to 1.25)	482 (285 to 750)	2.04 (1.2 to 3.17)	1045 (634 to 1621)	3.52 (2.12 to 5.51)	1.83 (1.69 to 1.98)
Portugal	2414 (1635 to 3440)	36.58 (24.61 to 52.33)	4768 (3372 to 6550)	54.78 (38.32 to 75.89)	1.31 (1.19 to 1.44)	217 (110 to 377)	3.29 (1.66 to 5.73)	474 (250 to 785)	5.41 (2.84 to 9.02)	1.62 (1.46 to 1.78)
Puerto Rico	823 (551 to 1168)	44.09 (29.56 to 62.46)	1536 (1075 to 2108)	60.13 (41.8 to 83.13)	1.03 (0.88 to 1.18)	65 (33 to 111)	3.5 (1.74 to 5.97)	139 (76 to 233)	5.44 (2.93 to 9.2)	1.51 (1.27 to 1.75)
Qatar	162 (102 to 244)	120.05 (78.33 to 177.06)	2298 (1499 to 3385)	173.7 (116.09 to 252.43)	1.18 (1.1 to 1.26)	12 (6 to 21)	7.96 (4.05 to 13.53)	163 (84 to 281)	11.6 (6.42 to 19.4)	1.24 (1.16 to 1.32)
Republic of Korea	7112 (4633 to 10247)	33.6 (22.18 to 47.98)	16544 (10994 to 23855)	34.35 (22.52 to 50.03)	0.08 (0.02 to 0.14)	575 (272 to 1000)	2.72 (1.28 to 4.7)	1243 (606 to 2154)	2.58 (1.25 to 4.49)	-0.14 (-0.25 to -0.03)
Republic of Moldova	1108 (719 to 1601)	42.37 (27.45 to 61.2)	1663 (1128 to 2276)	55.46 (37.37 to 76.37)	0.88 (0.81 to 0.95)	88 (43 to 154)	3.39 (1.64 to 5.94)	131 (68 to 222)	4.47 (2.29 to 7.6)	0.89 (0.76 to 1.01)
Romania	2339 (1416 to 3628)	14.43 (8.66 to 22.48)	2971 (1891 to 4440)	19.49 (12.33 to 29.2)	0.98 (0.94 to 1.03)	146 (75 to 244)	0.91 (0.46 to 1.53)	208 (104 to 358)	1.38 (0.69 to 2.39)	1.34 (1.24 to 1.44)
Russian Federation	18393 (11274 to 28443)	17.34 (10.58 to 26.84)	23658 (14536 to 36267)	19.97 (12.17 to 30.73)	0.45 (0.36 to 0.55)	1009 (535 to 1688)	0.97 (0.51 to 1.62)	1198 (631 to 2001)	1.02 (0.54 to 1.72)	0.19 (0.17 to 0.22)
Rwanda	823 (531 to 1197)	43.35 (28.14 to 62.75)	1974 (1263 to 2898)	43.8 (28.25 to 63.97)	0.02 (-0.06 to 0.11)	59 (31 to 98)	3.07 (1.61 to 5.07)	124 (64 to 213)	2.71 (1.41 to 4.64)	-0.4 (-0.55 to -0.25)
Saint Kitts and Nevis	7 (5 to 10)	48.19 (33.02 to 67.38)	28 (19 to 38)	58.9 (40.95 to 81.63)	0.65 (0.6 to 0.71)	1 (0 to 1)	4.25 (2.09 to 7.38)	3 (1 to 5)	5.64 (2.91 to 9.82)	0.93 (0.79 to 1.07)
Saint Lucia	34 (24 to 47)	74.05 (52.02 to 102.74)	119 (85 to 162)	86.96 (61.73 to 118.55)	0.53 (0.5 to 0.55)	3 (2 to 6)	7.66 (4.05 to 13.07)	13 (7 to 22)	9.38 (5 to 16.06)	0.65 (0.57 to 0.74)
Saint Vincent and the Grenadines	22 (15 to 30)	60.15 (41.78 to 83.68)	70 (50 to 95)	87.55 (62.56 to 119.91)	1.24 (1.2 to 1.27)	2 (1 to 4)	5.69 (3 to 9.8)	8 (4 to 13)	9.59 (5.12 to 16.53)	1.72 (1.62 to 1.83)

Samoa	16 (11 to 24)	30.49 (20.23 to 43.96)	60 (42 to 82)	64.03 (45.18 to 87.95)	2.43 (2.37 to 2.49)	1 (1 to 3)	2.74 (1.37 to 4.82)	7 (4 to 12)	7.5 (3.84 to 12.87)	3.32 (3.19 to 3.46)
San Marino	3 (2 to 4)	17.87 (10.69 to 28.25)	9 (6 to 13)	32.32 (21.29 to 46.84)	1.93 (1.89 to 1.98)	0 (0 to 0)	1.21 (0.59 to 2.14)	1 (0 to 1)	2.59 (1.27 to 4.39)	2.51 (2.38 to 2.64)
Sao Tome and Principe	11 (7 to 17)	29.99 (18.49 to 45.19)	30 (18 to 45)	37.3 (23.3 to 56.17)	0.7 (0.63 to 0.78)	1 (0 to 1)	1.84 (0.9 to 3.23)	2 (1 to 3)	2.2 (1.1 to 3.87)	0.61 (0.48 to 0.73)
Saudi Arabia	6403 (4120 to 9444)	148.2 (96.96 to 216.05)	35286 (24367 to 49150)	194.74 (136.11 to 267.94)	0.89 (0.85 to 0.93)	554 (281 to 968)	12.33 (6.54 to 21.09)	3006 (1614 to 4967)	15.66 (8.79 to 25.38)	0.77 (0.73 to 0.82)
Senegal	974 (643 to 1384)	47.54 (31.57 to 67.22)	3239 (2115 to 4668)	62.59 (41.08 to 89.91)	0.87 (0.78 to 0.95)	74 (37 to 125)	3.51 (1.78 to 5.96)	233 (117 to 396)	4.43 (2.23 to 7.52)	0.74 (0.55 to 0.94)
Serbia	1741 (1153 to 2566)	24.56 (16.19 to 36.3)	2530 (1746 to 3576)	36.4 (24.99 to 51.65)	1.27 (1.23 to 1.31)	142 (69 to 246)	2.04 (0.99 to 3.54)	240 (116 to 419)	3.51 (1.69 to 6.16)	1.78 (1.7 to 1.86)
Seychelles	14 (10 to 21)	49.07 (32.85 to 70.53)	107 (79 to 144)	140 (103.13 to 188.63)	3.49 (3.34 to 3.63)	1 (1 to 2)	3.66 (1.91 to 6.23)	12 (7 to 20)	16.42 (9.01 to 26.62)	5 (4.77 to 5.23)
Sierra Leone	172 (106 to 263)	14.66 (9.17 to 22.25)	644 (401 to 972)	24.74 (15.65 to 36.8)	1.72 (1.6 to 1.83)	10 (4 to 18)	0.83 (0.38 to 1.53)	38 (18 to 68)	1.41 (0.68 to 2.52)	1.79 (1.49 to 2.09)
Singapore	1027 (697 to 1451)	66.17 (45.35 to 92.86)	2419 (1586 to 3459)	50.45 (32.89 to 72.43)	-0.88 (-0.94 to -0.82)	93 (47 to 160)	5.96 (3.06 to 10.21)	184 (95 to 311)	3.84 (1.98 to 6.5)	-1.42 (-1.6 to -1.24)
Slovakia	533 (323 to 811)	17.04 (10.33 to 25.97)	966 (628 to 1440)	21.95 (14.19 to 32.81)	0.82 (0.79 to 0.84)	36 (19 to 60)	1.14 (0.6 to 1.94)	71 (37 to 123)	1.65 (0.84 to 2.85)	1.2 (1.08 to 1.32)
Slovenia	237 (147 to 364)	17.5 (10.84 to 26.9)	362 (231 to 544)	20.28 (12.83 to 30.58)	0.48 (0.42 to 0.54)	16 (8 to 27)	1.19 (0.59 to 2.03)	25 (12 to 43)	1.43 (0.7 to 2.47)	0.59 (0.51 to 0.68)
Solomon Islands	27 (18 to 40)	28.04 (18.57 to 40.44)	194 (136 to 268)	75.36 (53.21 to 103.62)	3.26 (3.16 to 3.36)	2 (1 to 4)	2.39 (1.15 to 4.21)	24 (12 to 42)	9.14 (4.68 to 15.73)	4.46 (4.32 to 4.6)
Somalia	568 (381 to 795)	27.46 (18.69 to 38.13)	1853 (1270 to 2553)	36.5 (25.4 to 49.74)	0.92 (0.9 to 0.95)	44 (25 to 72)	2.07 (1.15 to 3.37)	154 (75 to 265)	2.93 (1.45 to 5.04)	1.11 (1.06 to 1.16)
South Africa	6454 (4450 to 9047)	49.65 (34.35 to 69.44)	21764 (15378 to 30302)	73.22 (51.82 to 101.86)	1.27 (1.17 to 1.38)	538 (313 to 847)	4.05 (2.38 to 6.35)	1892 (1141 to 2949)	6.31 (3.83 to 9.81)	1.44 (1.19 to 1.7)
South Sudan	285 (178 to 419)	18.49 (11.76 to 26.91)	742 (476 to 1095)	23.81 (15.42 to 34.93)	0.83 (0.79 to 0.87)	17 (9 to 30)	1.11 (0.56 to 1.93)	46 (24 to 78)	1.48 (0.76 to 2.53)	0.93 (0.84 to 1.02)
Spain	16504 (11239 to 23480)	66.73 (45.2 to 95.23)	34094 (23540 to 47358)	89.1 (60.88 to 124.81)	0.94 (0.89 to 0.99)	1673 (859 to 2843)	6.78 (3.45 to 11.59)	3543 (1906 to 5821)	9.23 (4.91 to 15.29)	1 (0.95 to 1.06)
Sri Lanka	4141 (2725 to 6041)	58.33 (38.57 to 84.84)	17069 (12169 to 23662)	113.99 (81.13 to 158.2)	2.22 (2.04 to 2.4)	285 (149 to 479)	4 (2.09 to 6.72)	1529 (856 to 2524)	10.25 (5.69 to 16.98)	3.11 (2.86 to 3.35)
Sudan	4279 (2746 to 6270)	75.57 (49.01 to 110)	16326 (10986 to 23283)	118.01 (80.11 to 167.38)	1.47 (1.38 to 1.56)	296 (149 to 515)	5.12 (2.64 to 8.87)	1217 (670 to 2017)	8.62 (4.82 to 14.15)	1.69 (1.6 to 1.77)
Suriname	66 (42 to 99)	40.35 (25.53 to 60.01)	270 (187 to 377)	71 (49.04 to 99.32)	1.85 (1.82 to 1.88)	4 (2 to 7)	2.53 (1.27 to 4.32)	24 (12 to 41)	6.21 (3.26 to 10.68)	2.94 (2.75 to 3.14)
Sweden	637 (392 to 972)	11.52 (7.03 to 17.63)	1153 (748 to 1710)	15.79 (10.1 to 23.6)	1.02 (0.87 to 1.17)	56 (25 to 101)	1.01 (0.46 to 1.83)	105 (52 to 182)	1.43 (0.71 to 2.48)	1.14 (0.87 to 1.4)
Switzerland	923 (568 to 1412)	20.25 (12.43 to 31.04)	2074 (1359 to 3028)	28.85 (18.62 to 42.56)	1.14 (1.07 to 1.22)	64 (32 to 115)	1.41 (0.69 to 2.51)	157 (79 to 273)	2.17 (1.09 to 3.76)	1.4 (1.29 to 1.5)
Syrian Arab Republic	3450 (2217 to 5095)	97.4 (63.03 to 143.05)	10810 (7122 to 15686)	119.01 (78.11 to 173.21)	0.65 (0.62 to 0.67)	257 (132 to 449)	7.15 (3.72 to 12.44)	752 (399 to 1269)	8.29 (4.39 to 14.04)	0.48 (0.41 to 0.56)
Taiwan (Province of China)	3000 (1844 to 4506)	29.98 (18.45 to 44.96)	6923 (4621 to 10101)	31.27 (20.69 to 45.88)	0.12 (-0.04 to 0.28)	98 (46 to 179)	0.98 (0.46 to 1.79)	218 (100 to 396)	0.99 (0.45 to 1.81)	0 (-0.13 to 0.14)
Tajikistan	546 (333 to 822)	31.18 (19.11 to 46.82)	2382 (1468 to 3579)	52.74 (32.61 to 79)	1.71 (1.63 to 1.79)	31 (14 to 55)	1.75 (0.8 to 3.1)	131 (63 to 228)	2.88 (1.39 to 5)	1.62 (1.53 to 1.72)
Thailand	16267 (10128 to 24711)	65.49 (41.07 to 99.02)	48845 (33493 to 68213)	81.82 (55.54 to 115.36)	0.67 (0.49 to 0.85)	774 (405 to 1313)	3.12 (1.64 to 5.27)	2781 (1503 to 4574)	4.65 (2.5 to 7.68)	1.32 (1.12 to 1.52)
Timor-Leste	72 (46 to 106)	31.37 (20.31 to 46.17)	283 (192 to 399)	56.95 (38.93 to 80.19)	2 (1.82 to 2.19)	6 (3 to 11)	2.7 (1.24 to 4.85)	30 (14 to 53)	5.94 (2.82 to 10.48)	2.65 (2.48 to 2.82)

Togo	129 (78 to 197)	15.27 (9.38 to 23.07)	804 (481 to 1235)	28.25 (17.12 to 43.12)	2.02 (1.91 to 2.12)	7 (4 to 13)	0.85 (0.42 to 1.53)	46 (21 to 83)	1.58 (0.71 to 2.85)	2 (1.87 to 2.13)
Tokelau	0 (0 to 0)	30.45 (20.16 to 43.62)	0 (0 to 1)	60.82 (43.43 to 83.32)	2.27 (2.19 to 2.35)	0 (0 to 0)	2.7 (1.3 to 4.63)	0 (0 to 0)	6.99 (3.64 to 11.95)	3.14 (3.06 to 3.21)
Tonga	12 (9 to 15)	33.52 (25.1 to 43.66)	35 (25 to 48)	74.45 (53.46 to 100.44)	2.62 (2.56 to 2.68)	2 (1 to 3)	4.52 (2.17 to 7.69)	5 (3 to 9)	10.84 (5.82 to 18.09)	2.89 (2.78 to 3)
Trinidad and Tobago	320 (222 to 444)	67.16 (46.84 to 92.67)	756 (547 to 1015)	73.79 (53.2 to 99.6)	0.29 (0.22 to 0.36)	34 (18 to 58)	7.08 (3.8 to 12.13)	77 (41 to 129)	7.49 (3.95 to 12.69)	0.16 (0.05 to 0.28)
Tunisia	2169 (1412 to 3198)	68.45 (44.76 to 100.59)	8150 (5536 to 11752)	102.28 (69.39 to 147.59)	1.31 (1.27 to 1.35)	156 (77 to 277)	4.92 (2.42 to 8.7)	596 (329 to 979)	7.5 (4.13 to 12.34)	1.39 (1.26 to 1.53)
Turkey	11250 (7131 to 16904)	47.92 (30.5 to 71.78)	32936 (20925 to 49506)	60.28 (38.24 to 90.68)	0.72 (0.34 to 1.11)	701 (336 to 1254)	2.97 (1.43 to 5.3)	1809 (915 to 3207)	3.32 (1.68 to 5.88)	0.33 (-0.08 to 0.75)
Turkmenistan	362 (217 to 547)	28.8 (17.41 to 43.3)	1104 (664 to 1697)	39.12 (23.54 to 60.1)	0.98 (0.9 to 1.07)	19 (8 to 34)	1.49 (0.67 to 2.69)	57 (26 to 103)	2.02 (0.92 to 3.65)	0.99 (0.9 to 1.07)
Tuvalu	1 (1 to 2)	32.93 (21.74 to 46.93)	5 (3 to 6)	74.01 (52.5 to 101.58)	2.66 (2.58 to 2.73)	0 (0 to 0)	3.05 (1.47 to 5.38)	1 (0 to 1)	9.08 (4.75 to 15.39)	3.61 (3.36 to 3.85)
Uganda	1567 (1099 to 2135)	35.62 (25.21 to 48.23)	6946 (4682 to 9767)	62.08 (42.18 to 86.76)	1.84 (1.68 to 2)	151 (76 to 259)	3.35 (1.7 to 5.72)	602 (309 to 1046)	5.24 (2.76 to 9)	1.52 (1.3 to 1.74)
Ukraine	8542 (5327 to 12830)	21.68 (13.41 to 32.72)	9234 (5766 to 13709)	24.92 (15.43 to 37.24)	0.45 (0.43 to 0.48)	545 (256 to 946)	1.42 (0.67 to 2.47)	572 (270 to 1032)	1.58 (0.75 to 2.84)	0.36 (0.27 to 0.45)
United Arab Emirates	539 (348 to 803)	109.37 (72.99 to 158.58)	8842 (5935 to 12791)	139.89 (94.65 to 201.31)	0.8 (0.77 to 0.83)	45 (22 to 78)	8.24 (4.33 to 13.79)	712 (370 to 1212)	10.62 (5.84 to 17.54)	0.83 (0.79 to 0.86)
United Kingdom	12184 (8107 to 17498)	33.34 (22.09 to 48.01)	36308 (26689 to 48633)	72.46 (52.91 to 97.54)	2.54 (2.44 to 2.63)	921 (549 to 1435)	2.52 (1.5 to 3.94)	3570 (2292 to 5279)	7.11 (4.53 to 10.56)	3.42 (3.33 to 3.5)
United Republic of Tanzania	2431 (1495 to 3645)	34.48 (21.37 to 51.38)	8990 (5746 to 13258)	49.54 (31.93 to 72.67)	1.14 (0.94 to 1.34)	152 (72 to 272)	2.14 (1.01 to 3.81)	596 (311 to 1008)	3.24 (1.71 to 5.47)	1.29 (1.02 to 1.55)
United States of America	45688 (30174 to 65246)	31.75 (21.04 to 45.21)	112376 (80113 to 153322)	45.78 (32.26 to 62.93)	1.22 (1 to 1.44)	3685 (2146 to 5749)	2.57 (1.5 to 4)	10752 (6598 to 16210)	4.34 (2.64 to 6.57)	1.82 (1.33 to 2.31)
United States Virgin Islands	18 (12 to 27)	31.93 (20.37 to 46.97)	39 (27 to 55)	52.79 (36.26 to 74.71)	1.64 (1.58 to 1.7)	1 (1 to 2)	2.15 (1.12 to 3.65)	3 (2 to 6)	4.64 (2.42 to 7.93)	2.51 (2.41 to 2.61)
Uruguay	437 (261 to 684)	23.22 (13.77 to 36.51)	935 (572 to 1423)	40.55 (24.59 to 62.05)	1.8 (1.74 to 1.87)	19 (10 to 35)	1.02 (0.5 to 1.86)	41 (20 to 72)	1.77 (0.87 to 3.12)	1.78 (1.64 to 1.93)
Uzbekistan	1994 (1195 to 3066)	27.64 (16.64 to 42.31)	9128 (5636 to 13793)	48.11 (29.75 to 72.64)	1.8 (1.7 to 1.89)	112 (52 to 206)	1.55 (0.73 to 2.83)	488 (238 to 860)	2.57 (1.25 to 4.51)	1.68 (1.62 to 1.73)
Vanuatu	10 (6 to 15)	23.59 (15.07 to 34.3)	58 (41 to 81)	47.38 (33.13 to 66.33)	2.29 (2.22 to 2.37)	1 (0 to 1)	1.91 (0.96 to 3.32)	6 (3 to 10)	4.66 (2.42 to 8.07)	2.93 (2.8 to 3.05)
Venezuela (Bolivarian Republic of)	2524 (1619 to 3691)	40.06 (26.03 to 57.95)	10370 (7033 to 14737)	58.77 (39.76 to 83.7)	1.24 (1.15 to 1.33)	158 (81 to 265)	2.51 (1.3 to 4.18)	758 (401 to 1272)	4.3 (2.27 to 7.22)	1.76 (1.63 to 1.9)
Viet Nam	15027 (10282 to 21082)	64.82 (44.47 to 90.72)	67866 (50151 to 90895)	104.11 (76.88 to 139.51)	1.52 (1.35 to 1.7)	1107 (583 to 1821)	4.74 (2.49 to 7.8)	6262 (3479 to 10098)	9.61 (5.33 to 15.51)	2.31 (2.04 to 2.58)
Yemen	945 (609 to 1397)	27.66 (18.11 to 40.46)	5585 (3563 to 8426)	56.3 (36.56 to 84.38)	2.33 (2.23 to 2.44)	72 (32 to 134)	2.07 (0.92 to 3.83)	445 (211 to 805)	4.34 (2.13 to 7.7)	2.42 (2.23 to 2.62)
Zambia	584 (365 to 880)	29.54 (18.65 to 44.32)	1659 (1025 to 2534)	31.89 (19.9 to 48.31)	0.24 (0.21 to 0.27)	45 (22 to 79)	2.23 (1.08 to 3.9)	120 (59 to 206)	2.25 (1.11 to 3.86)	0.01 (-0.06 to 0.07)
Zimbabwe	916 (575 to 1385)	34.9 (22.14 to 52.39)	2295 (1449 to 3370)	47.07 (30.07 to 68.57)	0.93 (0.78 to 1.07)	81 (36 to 149)	3.06 (1.4 to 5.6)	192 (90 to 346)	3.88 (1.86 to 6.9)	0.74 (0.64 to 0.84)

AAPC, average annual percentage change; ASPR, age-standardized prevalence rate; ASYR, age-standardized YLD rate; YLDs, years lived with disability.

Within parentheses were 95% uncertainty intervals for numbers, ASPRs, and ASYRs, and 95% confidence intervals for AAPCs, respectively.
